# Extracellular vesicles released by steatotic hepatocytes alter adipocyte metabolism

**DOI:** 10.1002/jex2.32

**Published:** 2022-03-15

**Authors:** J.E. Mleczko, F. Royo, I. Samuelson, M. Clos‐Garcia, C. Williams, D. Cabrera, M. Azparren‐Angulo, E. Gonzalez, C. Garcia‐Vallicrosa, S. Carobbio, S. Rodriguez‐Cuenca, M. Azkargorta, S. van Liempd, F. Elortza, A. Vidal‐Puig, S. Mora, J.M. Falcon‐Perez

**Affiliations:** ^1^ Exosomes Laboratory Center for Cooperative Research in Biosciences (CIC bioGUNE) Basque Research and Technology Alliance (BRTA) Derio Bizkaia Spain; ^2^ Department of Neurology Alzheimer's Disease Research Center Icahn School of Medicine at Mount Sinai New York New York USA; ^3^ Centro de Investigación Biomédica en Red de enfermedades hepáticas y digestivas (CIBERehd) Instituto de Salud Carlos III Madrid Spain; ^4^ TVP Lab Wellcome/MRC Institute of Metabolic Science MRC Metabolic Diseases Unit – Metabolic Research Laboratories University of Cambridge Cambridge UK; ^5^ Novo Nordisk Foundation Center for Basic Metabolic Research (CBMR) Faculty of Health and medical Sciences University of Copenhagen Copenhagen Denmark; ^6^ Metabolomics Platform Center for Cooperative Research in Biosciences (CIC bioGUNE) Basque Research and Technology Alliance (BRTA) Derio Bizkaia Spain; ^7^ Proteomics Platform Center for Cooperative Research in Biosciences (CIC bioGUNE) Basque Research and Technology Alliance (BRTA) Derio Bizkaia Spain; ^8^ Department of Biochemistry and Molecular Biomedicine University of Barcelona Barcelona Spain; ^9^ IKERBASQUE Basque Foundation for Science Bilbao Bizkaia Spain

**Keywords:** adipocytes, exosomes, extracellular vesicles, hepatocytes, inflammation, liver steatosis, metabolic syndrome, microvesicles, obesity, Zucker model

## Abstract

The composition of extracellular vesicles (EVs) is altered in many pathological conditions, and their molecular content provides essential information on features of parent cells and mechanisms of crosstalk between cells and organs. Metabolic Syndrome (MetS) is a cluster of clinical manifestations including obesity, insulin resistance, dyslipidemia and hypertension that increases the risk of cardiovascular disease and type 2 diabetes mellitus. Here, we investigated the crosstalk between liver and adipocytes by characterizing EVs secreted by primary hepatocytes isolated from Zucker rat model, and studied the effect they have on 3T3‐L1 adipocytes. We found that steatotic hepatocytes secrete EVs with significantly reduced exosomal markers in comparison with their lean counterpart. Moreover, proteomic analysis revealed that those EVs reflect the metabolic state of the parent cell in that the majority of proteins upregulated relate to fat metabolism, fatty acid synthesis, glycolysis, and pentose phosphate pathway. In addition, hepatocytes‐secreted EVs influenced lipolysis and insulin sensitivity in recipient 3T3‐L1 adipocytes. Untargeted metabolomic analysis detected alterations in different adipocyte metabolic pathways in cells treated with hepatic EVs. In summary, our work showed that steatosis has a significant impact in the amount and composition of EVs secreted by hepatocytes. Moreover, our data point to the involvement of hepatic‐EVs in the development of pathologies associated with MetS.

## 1 INTRODUCTION

Metabolic syndrome (MetS) constitutes a cluster of diseases such as obesity, insulin resistance, hypertension and dyslipidemia that when occurring together predispose to more metabolic, severe complications such as T2DM, and cardiovascular diseases (Grundy, 2016). Obesity is one of the main factors leading to MetS and is a highly prevalent worldwide public health problem, projected to reach 365 million by the year 2030 (Grundy et al., 2005). The leading causes of obesity combine excessive nutrient intake and reduced physical activity with underlying metabolic or genetic susceptibilities (Myers et al., 2019). Obesity produces chronic low‐grade inflammation in major metabolic organs such as liver, adipose tissue and skeletal muscle, a central factor in the development of systemic insulin resistance (IR) (Longo et al., 2019).

EVs are a collective name given to membranous particles of various sizes released from cells to the extracellular milieu. Cells, both in vitro and in vivo secrete mixed populations of EVs with distinct structural and biochemical properties that circulate through the body in the different body fluids (Li et al., 2019). They are broadly classified based on their size and biogenesis, into exosomes, microvesicles (MVs) and apoptotic bodies. Furthermore, independently of their biogenesis, they are packaged with a broad range of molecules such as proteins, lipids, and nucleic acids: DNA and, both coding and noncoding RNAs (Doyle & Wang, 2019). Moreover, the cargo of EVs can be specific to their mode of biogenesis but also contains parental cell features. In that context, EVs have gained interest due to their role in cell‐to‐cell communication under both normal and pathological conditions. While delivering specific cargo, EVs can act in their local environment or be transported to distant tissues via endocrine system thereby regulating physiological processes (Yáñez‐Mó et al., 2015). They can transmit a wide range of stimuli, including cell proliferation, apoptosis, cytokine production, immune regulation, and metastasis (Hood et al., 2011; Peinado et al., 2011). An increasing number of recent reports indicate that overall, in pathological conditions the number of EVs being released is significantly elevated as compared to a healthy state, which can be monitored and explored for biomarker discovery (De Toro et al., 2015). Furthermore, emerging evidence supports the view that EVs play a role in the development of obesity and its complications (Dang et al., 2019; Gao et al., 2017). Thus, an increased number of circulating EVs has been detected in experimental rodent models of obesity but also in patients with T2D, hypertension, obesity and dyslipidemia (Martínez & Andriantsitohaina, 2017; O'Neill et al., 2016). Adipocyte‐released EVs impair insulin action in recipient cells, causing reduced AKT activation in cultured adipocytes (Mleczko et al., 2018) and hepatocytes (Kranendonk et al., 2014). Also, EVs isolated from *ob/ob* mice adipose tissue activate macrophages in vivo, resulting in an increased production of proinflammatory cytokines, interleukin‐6 and TNF‐α. Moreover, these EVs injected into wild‐type mice lowered glucose tolerance and increased insulin resistance (Deng et al., 2009). Remarkably, adipose‐derived EVs were able to increase the mTOR signalling in hypothalamic pro‐opiomelanocortin neurons leading to weight gain (Gao et al., 2020). All these reports highlight the importance of adipocyte‐secreted EVs in MetS development, but the involvement of hepatocyte‐secreted EVs has been underinvestigated. Recently, it was reported that EVs released by hepatocytes from high‐fat diet fed animals regulate lipid deposition and adipogenesis in cultured adipocyte cells (Zhao et al., 2020).

Here, we report a comprehensive and detailed molecular characterization of EVs secreted by hepatocytes in the context of MetS, using primary hepatocytes isolated from Zucker rats, a well‐established experimental model of MetS (Mleczko, 2018). Our data expands the knowledge on hepatocyte‐derived EVs and sheds light onto critical molecular aspects of cell‐cell communication processes with relevance to MetS. Furthermore, we show that hepatocyte‐released EVs represent novel effectors that might help to elucidate disease‐specific pathways in metabolic disease.

## 2 MATERIALS AND METHODS

We have submitted all relevant data of our EV‐related experiments to the EV‐TRACK knowledgebase (EV‐TRACK ID: EV210021) (Van Deun J., et al. EV‐TRACK: Transparent reporting and centralizing knowledge in extracellular vesicle research. *Nature Methods*. 2017;14(3):228–232).

### 2.1 Animal procedures

All animal experimentation was conducted in accordance with Spanish guidelines for the care and use of laboratory animals, and protocols were approved by the CIC bioGUNE Institute and the regional Basque Country ethical committee (ref. P‐CBG‐CBBA‐3610). All efforts were made to minimize the suffering of the animals. The surgery was performed under anesthetic gas Isoflurane (IsoFLO, Abbott Laboratories).

Male Zucker rats, Fatty (ZF) and its Lean (ZL) counterpart, (ZUC(Orl)‐*Lepr* ^fa^ strain) were purchased at 10–12 weeks of age from Charles River Laboratories (France). Animals were maintained in an environmentally controlled room at 22°C on 12 h light/dark cycle and provided with standard, 14% protein diet, (Rodent Maintenance Diet, Harlan Teklad Global Diet 2014) and water ad libitum.

### 2.2 Primary hepatocytes preparation

Primary hepatocytes were obtained by two‐step collagenase liver perfusion in 14 weeks old paired Zucker Lean and Zucker Fatty rats. Cell suspension was subjected to 90% Percoll™ (GE, Healthcare, NY) density gradient centrifugation (10 min at 500 rpm, 4°C) to increase the purity of hepatocyte population by excluding other liver cells. Cells were plated on collagen‐coated 150‐mm dishes, at 30 million cells per dish. Cells were cultured in complete DMEM medium [Dulbecco's modified Eagle medium supplemented with 10% (v/v) fetal bovine serum (FBS), 0.1 mg/ml streptomycin, and 100 units/ml penicillin (GIBCO, LifeTechnologies Inc.)] for 4 h, at 37°C and 5% of CO_2_. Cells were washed twice with Dulbecco's modified phosphate‐buffered saline (PBS) and incubated for 36 h in 25 mM HEPES containing complete DMEM medium (mostly depleted of contaminating vesicles though a 16‐h ultracentrifugation at 100,000 × *g*).

### 2.3 Culture and adipocyte differentiation of 3T3‐L1 cells

Mouse immortalised 3T3‐L1 cells were purchased from ATCC (CL‐173) and cultured in 10% CO_2_ at 370°C in growth media consisting of DMEM (4.5 g/L glucose; 25 mM), 10% calf serum and antibiotics, 1% penicillin (100 u/ml, Lonza), streptomycin (0.1 mg/L, Lonza, Walkersville, MD, USA). At 100% of confluence, fresh 10% calf media was replaced for additional 48 h. At 2 days postconfluence (day 0), cells were switched to differentiation media containing DMEM supplemented with, 10% FBS, 1 μg/ml bovine pancreas insulin (Sigma‐Aldrich), 0.25 μM dexamethasone (Sigma‐Aldrich), 500μM 1‐methyl‐3‐isobutyl‐xanthine (Sigma‐Aldrich) and 1 μM Rosiglitazone (Invitrogen) for 48 h. After 2 days, dexamethasone, 1‐methyl‐3‐isobutyl‐xanthine and Rosiglitazone were withdrawn, and cells are maintained in complete medium and 1 μg/ml insulin for another 48 h. At day 4, already differentiated adipocytes were maintained in DMEM with 10% FBS, and 1% PS. The medium was replenished every 2 days until day 12 when adipocytes become fully mature. For the Seahorse experiments, 3T3‐L1 cells were grown and differentiated on XF24 V7 microplates (Agilent Technologies) following the same protocol.

### 2.4 Isolation of EVs

EVs were isolated as previously described with minor modifications (Royo et al., 2013). Briefly, conditioned medium was centrifuged at 2000 × *g* for 10 min to remove dead cells and cellular debris. Resulting supernatant was filtrated through a 0.22 μm pore size filter system (Corning, NY USA) followed by sequential ultracentrifugation at 14,000 × *g* and 100,000 x *g* for 30 min and 75 min, respectively (45Ti rotor in Optima L‐100 XP, Beckman Coulter Inc.). Pellets obtained after 100,000 x *g* were resuspended in 1XD‐PBS and ultracentrifuged again at 100,000 × *g* 4°C during 75 min. The final pellets were resuspended in 150 μl 1XD‐PBS, aliquoted into 30 μl aliquots and stored at −80°C.

### 2.5 Cryo‐electron microscopy

EV samples were used for cryo‐electron microscopy, which was performed as previously described (Royo et al., 2013). Briefly, for cryo‐electron microscopy, EV preparations were directly adsorbed onto glow‐discharged holey carbon grids (QUANTIFOIL, Germany). Grids were blotted at 95% humidity and rapidly plunged into liquid ethane with the aid of VITROBOT (Maastricht Instruments BV, The Netherlands). Vitrified samples were imaged at liquid nitrogen temperature using a JEM‐2200FS/CR transmission cryo‐electron microscope (JEOL, Japan) equipped with a field emission gun and operated at an acceleration voltage of 200 kV.

### 2.6 Nanoparticle tracking analysis (NTA)

Particle size distribution and concentration were analysed by measuring the rate of Brownian motion using a NanoSight LM10 system (NanoSight, Amesbury, U.K.). The system is equipped with a fast video capture and particle‐tracking software. NTA post acquisition settings were kept constant for all samples, and each video was analysed to give the mean, mode, and median vesicle size, and the concentration of vesicles in a given sample. 1 μg of EV samples was analysed and the dilution factor used for normalization. Each EV preparation was determined by triplicate.

### 2.7 Iodixanol density gradient

Hepatocyte‐secreted EVs were also characterized in iodixanol density gradient (OptiPrep™, Sigma‐Aldrich). Briefly, a discontinuous iodixanol gradient was established by preparing 5% and 40% dilutions of the original OptiPrep™, 60% w/v stock in ice‐cold 20 mM HEPES pH 7.4 solution. The gradient was set up in polyallomer tube (Beckman Coulter Cat. No. 344059) by subsequent layering of 5.5 ml fractions of 40% and 5% iodixanol solution. Equal amount of EVs (250 mg) was layered on top of a 5% iodixanol and centrifuged at 100,000 x *g* for 4 h in no break mode for stopping speed (SW40Ti rotor, Beckman Coulter Inc.). Using Autodensity‐flow 230 V (Cat No 4517200, Labconco), 12 individual 1 ml fractions of increasing density were collected. The refractory index (RI) was measured by means of refractometer (Abbe 2WAJ). RI was translated to density following the correlation built from the data provided by OptiPrep™. All fractions were diluted in 20 mM Hepes pH 7.4 and centrifuged at 100,000 x *g* for 1 h at 4°C. The resulting pellets were each resuspended in 30 μl of sterile PBS.

### 2.8 Western blot analysis

Cells (1 × 10^6^) were lysed on ice with 150 μl of the lysis buffer (1% Triton X‐100, 300 mM NaCl, 50 mM Tris‐HCl, pH 7.4). The protein concentration of cell extract and EVs was determined by Bradford protein assay using BSA as standard, and 10 μg of cell lysate and 5 μg of EVs were loaded per lane. The samples were prepared in NuPAGE LDS 4X Sample Buffer (Life Technologies Inc.) and sequentially heated up for 5 min at 37°C, 65°C, and 95°C before being separated on 4%–12% precasted gels (Thermo Fisher Inc.). Gels were transferred onto a Polyvinylidene difluoride (PVDF) membrane using Life Technologies iBlot^®^ 2 Dry Blotting System (program 0: 20 V for 1 min, 23 V for 4 min and 25 V for 2 min, default run time being 7 min) or onto nitrocellulose membranes by electroblotting using a Mini Trans–Blot cell (Bio‐Rad). Membranes were blocked with 5% blotting‐grade blocker (BioRad) in 1XPBS containing 0.1% Tween‐20 (Sigma) for 1 h at room temperature (RT), washed three times with T‐PBS‐0.1% and incubated overnight 4°C with commercial primary antibodies. Membranes were then washed with 1X T‐PBS and incubated for 1 h at RT in blocking solution‐containing secondary antibodies conjugated to horseradish‐peroxidase (HRP). All proteins were detected under nonreducing conditions. Chemiluminescence detection of bands was performed using ECL Plus reagent (GE Healthcare, Buckinghamshire,UK). Protein bands were quantified by densitometry analysis using ImageJ software. The complete list of primary antibodies can be found in Table S1.

### 2.9 Proteomics analysis

#### 2.9.1 Tryptic digestion of gel bands

Fifty microgram of sample was loaded on a gel and after a short electrophoretic running, gel bands were cut and washed in milli‐Q water. Reduction and alkylation were performed using dithiothreitol (10 mM DTT in 50 mM ammonium bicarbonate) at 56°C for 20 min, followed by iodoacetamide (50 mM iodoacetamide in 50 mM ammonium bicarbonate) for another 20 min in the dark. Gel pieces were dried and incubated with trypsin (12.5 μg/ml in 50 mM ammonium bicarbonate) for 20 min on ice. After rehydration, the trypsin supernatant was discarded. Gel pieces were hydrated with 50 mM ammonium bicarbonate, and incubated overnight at 37°C. After digestion, acidic peptides were cleaned with TFA 0.1% and dried out in a RVC2 25 SpeedVac concentrator (Christ). Peptides were resuspended in 10 μl 0.1% FA and sonicated for 5 min prior to analysis.

#### 2.9.2 Protein extraction

An equivalent volume of a solution containing 7 M Urea 2 M Thiourea 4% CHAPS 100 mM DTT was added to the samples for protein extraction, and incubated under agitation for 30 min. The sample was vortexed for 30 s every 10 min of incubation.

#### 2.9.3 FASP in solution digestion and sample preparation

In solution digestion was carried out following FASP protocol with minor variations (Wiśniewski et al., 2009). This protocol relies in the use of standard filtration devices, allowing buffer exchange and acting as a reactor for the digestion. Twenty minute centrifugations at 13,000 rpm are carried out between each digestion step in order to remove buffer from the filter. Samples were loaded onto Amicon Ultra 0.5 ml 30 K centrifugal units (Millipore), washed twice in UA solution (8 M Urea, 100 mM Tris‐HCl pH 8.5) and alkylated by a 20 min incubation in 50 mM Chloroacetamide prepared in UA solution. Three more washes in UA were carried out, followed by additional three washes in 50 mM AMBIC. Protein was quantified using Bio‐Rad protein assay (Bio‐Rad), and trypsin was added to a trypsin:protein ratio of 1:10. The mixture was incubated overnight at 37°C. Peptides were recovered from the filter units and subjected to ethyl acetate extraction. Briefly, 1 ml of water‐saturated ethyl acetate was added to the peptide solution and vortexed for 1 min. Then, tubes were centrifuged 15 s at 13,000 rpm and the upper ethyl acetate layer was depleted. These steps were repeated 5 times. After the careful removal of the last upper ethyl acetate layer, samples were subjected to speed‐vacuum in a RVC2 25 SpeedVac concentrator (Christ). Samples were further desalted using stage‐tip C18 microcolumns (Zip‐tip, Millipore) and peptides were resuspended in 0.1% FA prior to MS analysis.

#### 2.9.4 Mass spectrometry analysis

Peptide separation was performed on a nanoACQUITY UPLC System (Waters) connected to an LTQ Orbitrap XL mass spectrometer (Thermo Electron) or a Synapt G2 Si (Waters). An aliquot of each sample was loaded onto a Symmetry 300 C18 UPLC Trap column (180 μm × 20 mm, 5 μm (Waters). The precolumn was connected to a BEH130 C18 column, 75 μm x 200 mm, 1.7 μm (Waters), and equilibrated in 3% acetonitrile and 0.1% FA. Peptides were eluted directly into the nanoelectrospray capillary (Proxeon Biosystems) at 300 nl/min, using a 60 min linear gradient of 3%–50% acetonitrile. The LTQ Orbitrap XL ETD automatically switched between MS and MS/MS acquisition in DDA mode. Full MS scan survey spectra (*m*/*z* 400–2000) were acquired in the orbitrap with mass resolution of 30,000 at *m*/*z* 400. After each survey scan, the six most intense ions above 1000 counts were sequentially subjected to collision‐induced dissociation (CID) in the linear ion trap. Precursors with charge states of two and three were specifically selected for CID. Peptides were excluded from further analysis during 60 s using the dynamic exclusion feature.

#### 2.9.5 Database searches

Mascot search engine v2.1 (Matrix Science) through Proteome Discoverer 1.4. (Thermo Electron). Carbamidomethylation of cysteines was set as fixed modification, and oxidation of methionines as variable modification, and two missed cleavages were allowed. Ten ppm of peptide mass tolerance and 0.5 Da fragment mass tolerance were used for Orbi acquisitions, whereas 15 ppm peptide mass tolerance and 0.2 Da fragment mass tolerance was used for Synapt G2Si runs. Spectra were searched against *Rattus norvegicus* or *Mus musculus* databases obtained from Uniprot/Swissprot (database version 2016_03). A decoy search was carried out in order to estimate the false discovery rate (FDR). Only peptides with a false discovery rate of <% were selected.

#### 2.9.6 Progenesis LC‐MS software analysis

Progenesis LC‐MS (version 4.0.4265.42984, Nonlinear Dynamics) was used for the label‐free differential protein expression analysis. After importing of the Raw files, one of the runs was used as the reference to which the precursor masses in all other samples were aligned to. Only features comprising charges of 2+ and 3+ were selected. The raw abundances of each feature were automatically normalized and logarithmised against the reference run. Samples were grouped in accordance with the comparisons being performed, and an ANOVA analysis was performed. A peak list containing the information of the features was generated and exported to the Mascot search engine (Matrix Science Ltd.) and searched against the database. The list of identified peptides was imported in Progenesis LC‐MS and the previously and the three most intense nonconflicting peptides (peptides occurring in only one protein) of each protein were specifically chosen for quantitative purposes. Proteins with at least two different nonconflicting peptides and a *p* value < 0.05 were selected for further analyses.

#### 2.9.7 Functional analysis

GO enrichment analysis was carried out using the DAVID online tool (http://david.abcc.ncifcrf.gov/summary.jsp) (Huang et al., 2009). DAVID is a GO Term annotation and enrichment analysis tool used to highlight the most relevant GO terms associated with a given gene list. A Fisher Exact test is used in order to determine whether the proportion of genes considered into certain GO terms or categories differs significantly between the dataset and the background. An FDR‐corrected version of the Fisher's test *p*‐value can be obtained and used for more conservative result selection. Biological Process (BP), Molecular Function (MF) and Cellular Component (CC) categories were assessed, and only GO Terms enriched with FDR < 5% were considered for comparison and discussion. Additionally, KEGG Pathways was also analysed, considering terms with an enrichment *p*‐value < 0.01. In addition, DAVID provides a Functional Annotation Clustering report, where similar annotations are displayed together, making the interpretation of the result biology clearer and more focused in enriched processes. The Group Enrichment Score gives the geometric mean (in‐log scale) of the cluster member's *p*‐values, and is used to rank their biological significance.

### 2.10 Deoxy‐glucose uptake assay

Insulin‐stimulated 2‐deoxyglucose assay was determined in fully differentiated 3T3L1 adipocyte cells as we previously described (Mleczko et al., 2018). Briefly, before the glucose uptake assay, cells were incubated in DMEM media alone or containing EVs as indicated in the figure legends for 24 h. Cells were washed in Krebs Ringer Hepes buffer (pH = 7.4) twice and incubated in this buffer for 45 min. Insulin was added at 100 nM for 30 min prior to the assay. Two‐deoxyglucose uptake media was added containing 2μCi/ml of ^3^H‐2‐deoxyglucose and unlabelled substrate at 0.1 mM final concentration. Uptake was allowed to proceed for 10 min and stopped by washing four times in ice cold PBS containing 50 mM glucose. Cells were lysed in 0.1 N NaOH and a fraction of lysate counted in scintillation cocktail. An aliquot of lysate was used for protein determination. All transport measurements were normalized to protein content in the cellular lysates.

### 2.11 BODIPY™ fluorescent staining

Cells were grown and differentiated over 12 mm glass coverslips and fixed with 2% paraformaldehyde (Santa Cruz Biotechnology). Fixed cells were stained with 10 μg/ml of BODIPY^R^493/503 (4,4‐Difluoro‐1,3,5,7,8‐Pentamethyl‐4‐Bora‐3a,4a‐Diaza‐*s*‐Indacene (Molecular Probes, Invitrogen) diluted in PBS for 45 min at room temperature protected from light. Following two times‐10 min washes in PBS, cells were imaged using Axioimager D1 microscope (Zeiss). Protocol modified from Gocze and Freeman (1994).

### 2.12 Lipolysis assay

Lipolysis was measured by detection of free glycerol in cell medium upon stimulation with 10 μM norepinephrine (NE) for 2 h. Fully differentiated 3T3‐L1 cells in 96‐well plates were incubated overnight without (PBS) or with 5 μg/well of EVs (ZL or ZF) in serum‐free medium without phenol red, after which cells were stimulated with 10 μM NE for 2 h. Medium was collected and glycerol content measured against a standard curve of glycerol using free glycerol reagent (Sigma‐Aldrich) and an Epoch™ two microplate reader. Glycerol values were normalized to DNA content (DAPI).

### 2.13 Metabolic extracellular flux assay

Cellular metabolic ECAR profile, reflecting the rate of glycolysis in 3T3‐L1 adipocytes treated with ZL‐ or ZF‐hepatocytes derived EVs was determined, in real time, using the glycolysis stress test and a Seahorse XF24 Extracellular Flux Analyzer (Agilent Technologies). 3T3‐L1 adipocytes were allowed 4 h to adhere and subsequently incubated in humidified incubator without CO_2_ for 1 h before the assays in unbuffered medium (DMEM, 8.3 g/L, Sigma). The glycolysis stress test requires the sequential injection of glucose (40 μl/ml of 100X), oligomycin (2 μl/ml of 10 mg/ml) and finally 2‐Deoxy‐D‐glucose [(2‐DG) 0.18 g/ml, pH 7.4]. The measurements were normalized to cellular content estimated by crystal violet. Briefly, cells were fixed and stained with 0.1% crystal violet stain in 20% methanol solution for minimum 1 h at RT. Next, wells were washed twice with distilled water to eliminate the excess crystal violet. Once the wells were completely dry, a 10% acetic acid solution (50 μl) was added to collect the crystal violet. The absorbance at 595 nm wavelength was determined by spectrophotometry using a Spectramax M3 instrument.

### 2.14 Untargeted metabolomics analysis

For extraction 500 μl of a mixture of ice‐cold methanol/water (50/50% v/v) with 10 mM acetic acid was added to the wells of the 12‐wells culture plates. The plates were left on dry ice for 15 min. Subsequently, 400 μl of the homogenate plus 400 ul of chloroform was transferred to a new aliquot and shaken at 1400 rpm for 30 min at 4°C. Next, the aliquots were centrifuged for 30′ at 14000 rpm at 4°C. Seventy‐five microliters of the aqueous phase was transferred to a fresh aliquot and placed at −80°C for 20′. The chilled supernatants were evaporated with a SpeedVac in approximately 2 h. The resulting pellets were resuspended in 100 μl water/acetonitrile (MeCN (40/60/ v/v/). Short and chronic treatments were analysed separately, following the same pipeline. Three pairwise comparisons were performed per each treatment: control versus lean, control versus obese and lean versus obese.

Firstly, data was cleaned‐up, removing all those features that were presented with more than 30% of missing values. Missing values of the remaining metabolites were imputed by taking the 10% of minimal value of the corresponding feature. With the data cleaned, Venn diagrams were obtained to identify which metabolic features were present in both groups compared and which ones were specific of a certain group with VennDiagram R package. We then computed the fold change for each feature, and we applied *t*‐student's test to identify which alterations were statistically significant. *P*‐values were adjusted for multitesting with Bonferroni methodology. Volcano plots were generated to facilitate the interpretation of results. Supervised multivariate analysis was performed using metabolomics features. Differential peaks were used to identify specific metabolites with METLIN database. The levels of the 13 identified metabolites were represented by using boxplots indicating the *t*‐student's *p*‐value per each pairwise comparison and the ANOVA *p*‐value for the comparison of the three groups (control, lean and obese).

### 2.15 Statistical analysis

Data calculation and statistical analysis were performed using GraphPad Prism 6.0 software. The statistical significance of difference between two groups was determined with unpaired Student's *t* test and multiple comparisons were analysed using one‐way ANOVA. Differences were considered statistically significant at *p < *0.05.

## 3 RESULTS

### 3.1 Molecular characterization of EVs secreted by primary hepatocytes from the Zucker rat model

**FIGURE 1:**
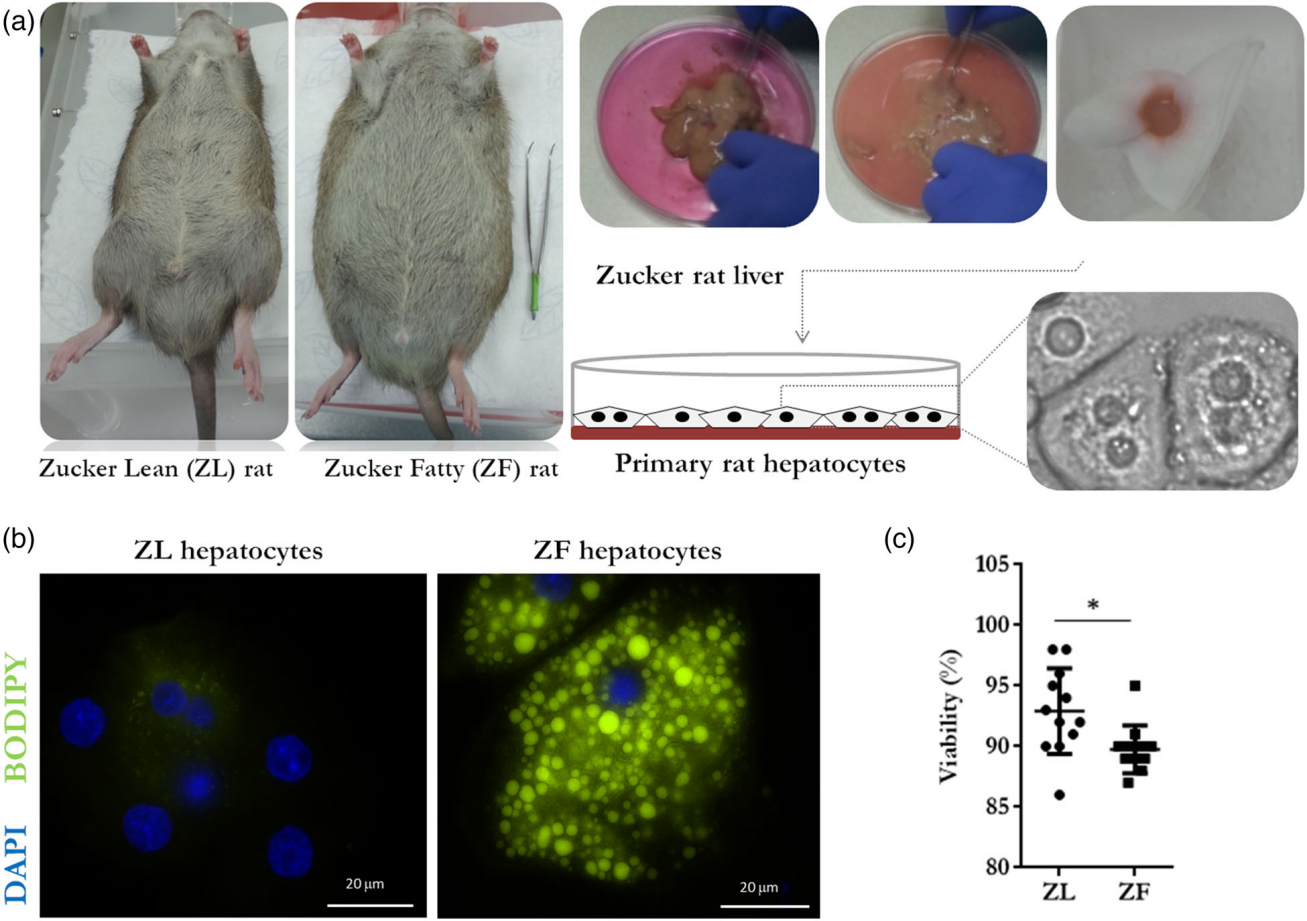
Primary hepatocytes from Zucker rat model. (a) Representative photographs of 10‐weeks old heterozygous Zucker lean (+/*fa*; ZL) and homozygous (*fa*/*fa*; Zucker fatty; ZF) rats, and of the main steps of the two step collagenase perfusion technique including manual dissociation and gauze filtration before seeding the cells into the collagenized‐plates. *Note*: Isolation and quality of primary hepatocytes obtained using this procedure are supported by the representative image taken under visible microscope. (b) Representative images of bodipy lipid staining of ZL‐ and ZF‐hepatocytes after 4 h in culture; size bar = 20 μm. (c) Cell viability assessed by trypan‐blue exclusion method; *n* = 12. The *p* values were denoted as follows: ns *p* > 0.05, * *p* ≤ 0.05, ** *p* ≤ 0.01, *** *p* ≤ 0.001, **** *p* ≤ 0.0001

Obese (*fa*/*fa*) Zucker rat is a genetic model of obesity, which allows the study of its complications and effects on other organs in the body (Lutz & Woods, 2012). In the current study, primary hepatocytes, from livers of obese rat *fa*/*fa* (ZF) and its genetic lean counterpart *fa*/*FA* (ZL), were isolated and cultured for the study of EVs released into conditioned media (Figure 1a). To confirm that the primary cells maintained their steatotic phenotype in culture, the lipophilic dye BODIPY^R^ was used to stain intracellular fat droplets in primary hepatocytes. Fat droplet content was detected in steatotic hepatocytes, as compared to the control (Figure 1b). Cell viability was determined by Trypan blue exclusion method and showed overall viability ranging between 86% and 98% in 12 independent paired‐isolations (Figure 1c). Remarkably, in both lean and obese models, viability of hepatocytes was higher than 85% supporting the good quality of the primary cultures.

**FIGURE 2:**
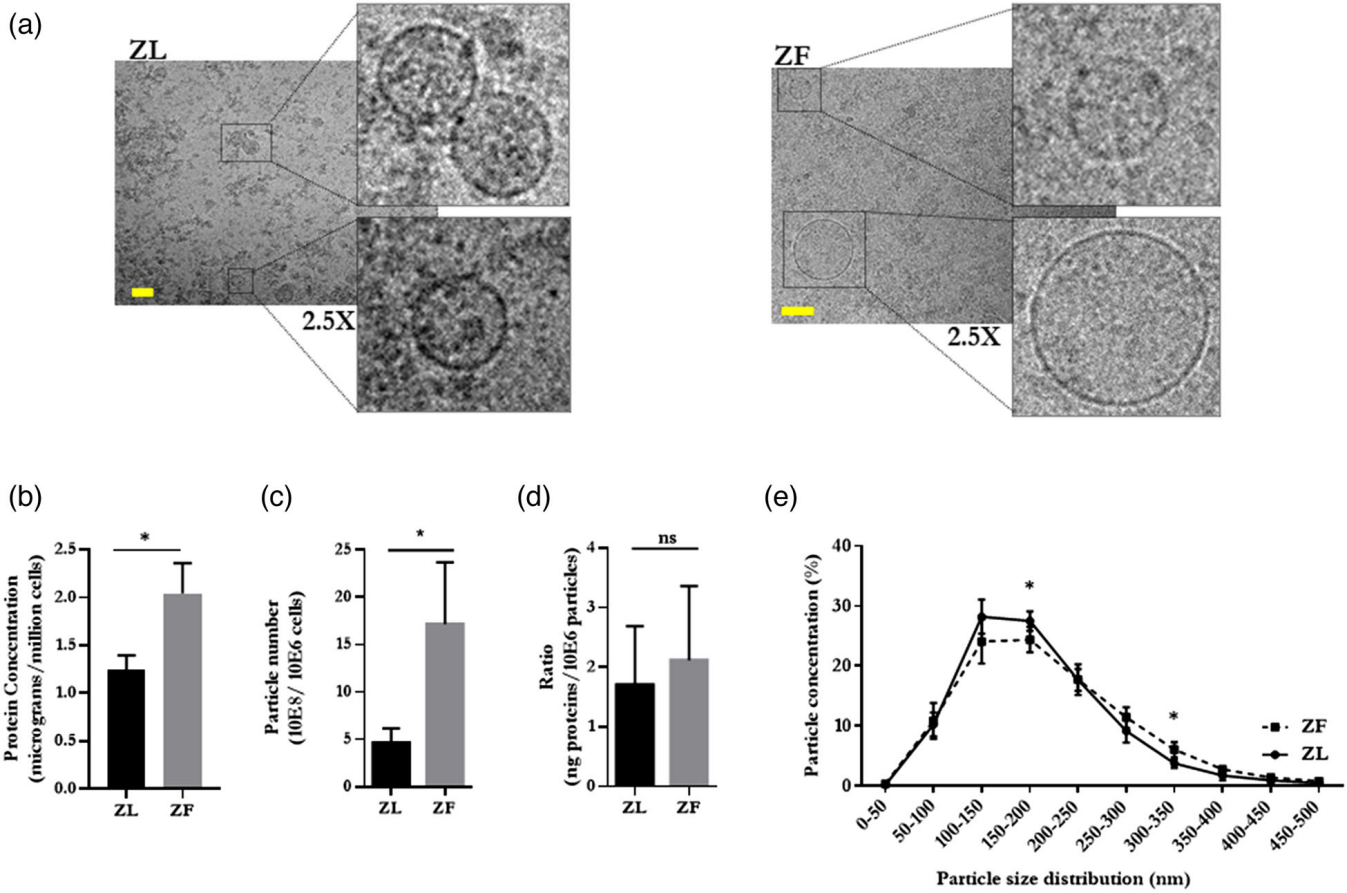
Ultrastructural and biochemical characterization of EVs isolated from conditioned medium of ZL and ZF hepatocytes. (a) Representative cryo‐electron micrographs of ZL‐ and ZF‐EVs; size bar = 100 nm; inserts magnified 2.5×. (b) Protein concentration of the EVs preparations measured by Bradford assay; *n* = 12. Each EV preparation was done from 300 millions of cells. (c) Particle number measured by NTA; *n* = 12. Each EV preparation was done from 300 million of cells. (d) Ratio per million of cells of protein concentration versus particle number; *n* = 4. (e) Size range distribution measured by NTA; *n* = 4. Error bars = SD. Significance was determined by *t*‐test between the two groups. The *p* values were denoted as follows: ns *p* > 0.05, * *p* ≤ 0.05, ** *p* ≤ 0.01, *** *p* ≤ 0.001, **** *p* ≤ 0.0001

EVs were isolated from the conditioned media of primary ZL and ZF hepatocytes, following a 36 h culture period in EV‐depleted media. Cryo‐electron microscopy revealed that the population of hepatocyte‐derived EVs consisted primarily of round, typically translucent and enclosed in a visible, electron‐dense lipid bilayer, in both the ZL and ZF samples (Figure 2a); consistent with previously published data (Conde‐Vancells et al., 2008). Subsequently, we analysed the average protein content in EVs obtained from 12 independent ZL‐ and ZF‐EVs paired‐preparations (Figure 2b). We detected a nearly two‐fold increase in the protein content in EV preparations from ZF hepatocytes compared to those released by their lean counterpart (Figure 2b), suggesting an increased number of protein‐containing particles secreted from steatotic hepatocytes. This was confirmed by NTA, where a significant increase in particle number was observed in preparations from ZF hepatocytes (Figure 2c). We also determined the ratio between protein content and the number of particles as an indication of the purity of EVs and consistency between independent preparations (Webber & Clayton, 2013). Our preparations were reproducible with the average ratios of 1.7 (±0.5) and 2.1 (±0.6) for ZL‐ and ZF‐EVs, respectively (Figure 2d).

Interestingly, NTA analysis demonstrated changes in the size distribution of particles secreted by ZL‐ and ZF‐ primary hepatocytes. Although in both cases, the majority (>90%) of particles in our EV preparations after differential ultracentrifugation (at 10,000 and 100,000 × *g*, see Material and Methods section) were below 300 nm in diameter (Figure 2e), there was a significant decrease in 150–200 nm size range and a concomitant increase in larger vesicles (300–350 nm) in preparations obtained from ZF hepatocytes (Figure 2e).

The increased protein abundance and different size distribution of the particles released by ZF hepatocytes suggested the possibility that steatosis results in the production of different populations of EVs with potentially distinct physiological effects in recipient cells.

### 3.2 Obese primary hepatocytes release differential subpopulations of EVs

**FIGURE 3:**
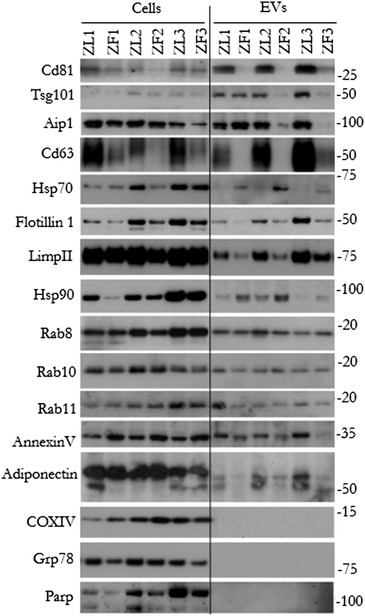
Western‐blot analysis of Zucker rat hepatocyte‐secreted EVs. Protein extracts from ZL‐ and ZF‐primary hepatocytes (10 μg) and their corresponding EVs (5 μg) were analysed by Western‐blotting using antibodies against the indicated proteins; *n* = 3

The differences observed in protein content and size distribution between the EVs secreted by ZL and ZF‐hepatocytes prompted us to evaluate if there were also changes in the protein cargo. Firstly, we assessed the protein size distribution by SDS‐PAGE gel followed by Sypro Rubi staining. No significant differences were observed in the pattern of protein distribution between samples (Figure S1). Next, three independent paired‐preparations of EVs along with their corresponding cell extracts were analysed by Western blotting for well‐known EV marker proteins (Figure 3). Remarkably, while the Cd81 and Cd63 were detected in EVs secreted by ZL hepatocytes, these proteins were barely detected in EVs from steatotic ZF hepatocytes. Other markers such as ESCRT machinery associated proteins (Tsg101, Alix), lipid‐raft associated protein (Flotilin1), the heat shock protein of 90 kDa, (Hsp90), the lysosomal and late endosomal membrane protein, LimpII were detected in both samples albeit in significantly reduced levels in the EVs from the steatotic model (Figure 3). Concomitantly a similar trend was observed for the cellular lysates, supporting the idea that EV cargo composition resembles that of the parental cells. Interestingly, we observed higher abundance of Hsp70 and Hsp90 in EVs derived from ZF hepatocytes, suggesting a preferential loading and export of these proteins into EVs in ZF hepatocytes. In the context of signalling in metabolic syndrome, we also detected the presence of adiponectin in our EV preparations with levels being significantly reduced in ZF‐EVs (Figure 3). Notably, the protein markers for subcellular compartments such as mitochondrial CoxIV and ER‐associated Grp78 were clearly detected in cellular extracts but were absent in EV preparations (Figure 3), indicating that in our culture conditions contaminating‐cellular lysis was not generated. This was also supported by the fact that there was no significant apoptosis in either of the two models, as judged by the pattern seen for the apoptotic marker PARP (Figure 3).

**FIGURE 4:**
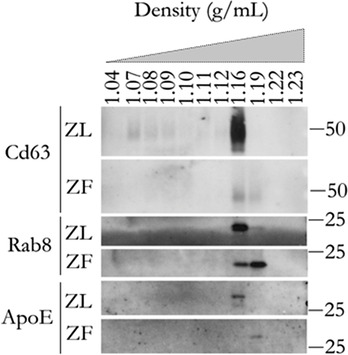
Density gradient analysis of EVs secreted by hepatocytes. Based on their density, EVs were separated by ultracentrifugation in a density gradient of idioxanol. Fractions were analysed by Western blotting using antibodies against the indicated proteins

Considering the differences observed, we further characterized the EVs by analysing their flotation in a density gradient (Figure 4). Interestingly, Western blot analysis revealed that while Cd63, Rab8 and ApoE in ZL‐EV sample is associated to a single fraction, at a density of 1.16 g/ml, the ZF‐derived EVs were detected in two fractions, of 1.16 and 1.19 g/ml densities. Significantly, higher expression of Rab8 and ApoE proteins were associated with denser fraction in ZF‐EV sample, as compared to the control (Figure 4).

These data suggest that under steatotic conditions hepatocytes secrete a higher number of EVs, and with an altered protein cargo.

### 3.3 Proteomic profiling of EVs

To gain further insight into the protein signature of EVs preparations obtained from steatotic hepatocytes, we performed a proteomic mass spectrometry analysis where we detected a total of 438 proteins (Table S2). A gene ontology classification of these proteins revealed that almost 50% belong to the category of catalytic activity of molecular function, and almost 30% to the category of the metabolic process of biological process (Table S3). A more detailed analysis showed that many metabolic pathways including glycolysis, pentose phosphate, TCA, oxidative phosphorylation and lipid metabolism were represented in the EV protein cargo as judged by the KEGG database (Figure S2a). All these results together suggest the involvement of hepatocyte‐secreted EVs in these metabolic pathways.

**FIGURE 5:**
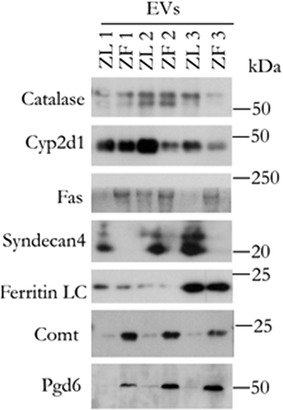
Western‐blot analysis of DEPs identified by quantitative label free proteomic analysis. Three independent preparations of EVs from Lean and Obese Zucker rat hepatocytes (10 μg) were analysed by Western blotting by using antibodies against indicated proteins

The comparison of the protein‐cargo of EVs secreted by ZL and ZF hepatocytes indicates that 145 (33% of the total) were significant and differentially expressed proteins (DEPs). Among them, 35 and 110 proteins were up‐ and down‐regulated, respectively in ZF EVs compared to ZL EVs (Table S2 and Figure S2b). The proteomics data confirmed some of the results obtained previously such as a reduction in ZF‐EVs of the EV marker proteins Cd81 or Aip1. Also, we further confirmed the proteomics data, by performing additional Western‐blotting of some of the up‐ and down‐regulated DEPs (Figure 5). In agreement with the proteomics data, increased expression in Fas, Comt and Pgd6 enzymes was observed in ZF‐EVs. Moreover, Catalase, Cyp2d1, Syndecan 4 as well as Ferritin LC were found to be down‐regulated in ZF‐EVs, as indicated also by the proteomics data.

From a metabolic point of view, several enzymes involved in glycolysis were augmented in ZF‐EVs (Table 1 and Figure S3). Also, oxidative phase enzymes of the Pentose Phosphate Pathway (PPP), phosphogluconate dehydrogenase (6PGD) and glucose‐6‐phosphate 1‐dehydrogenase (G6PD) was profoundly upregulated in EVs secreted by steatotic hepatocytes (Table 1 and Figure S4). Besides, enzymes belonging to the TCA cycle and oxidative phosphorylation were also detected in EVs and some of them significantly altered in EVs from steatotic hepatocytes (Table 1 and Figure S5a,b). Most processes associated with lipid metabolism are predominantly performed in the liver, including synthesis of lipoproteins, fatty acids, and triglycerides. Accordingly, many enzymes related to lipid metabolism were detected in EVs, comprising the second most enriched group of upregulated proteins found in ZF EVs (Table 1 and Figure S6a,b). Among these we detected fatty acid synthase (Fas) and acetyl‐CoA carboxylase 1 (Acaca) enzymes, both crucial for long‐chain fatty acid formation. Among the DEPs that were significantly reduced in EVs secreted by steatotic hepatocytes we found the glucose transporter 2 (Glut2) and several members of P450 superfamily and glucuronosyltransferases (UGTs). Interestingly, 40% of downregulated proteins were related to mitochondria (Table 1).

**TABLE 1:** Analysis of differentially expressed proteins in EVs secreted by primary hepatocytes from Zucker rat model

					CATEGORIES					
Protein ID	Protein ID	Log (FC) obese/lean	Anova (*p*)	Protein description	Catalytic activity	Fat metabolism	Mitochondria	The citric acid circle and electron transport	Pentose phosphate pathway	Pyruvate metabolism
G6PD_RAT	P05370	3.95	6.05E‐04	Glucose‐6‐phosphate 1‐dehydrogenase	✓	✓	✗	✗	✓	✗
MAOX_RAT	P13697	2.85	2.09E‐05	NADP‐dependent malic enzyme	✓	✓	✗	✗	✗	✓
RL23_RAT	P62832	2.73	5.23E‐03	60S ribosomal protein L23	✗	✗	✗	✗	✗	✗
ST2A2_RAT	P22789	2.35	3.78E‐03	Alcohol sulfotransferase A	✓	✗	✗	✗	✗	✗
ACLY_RAT	P16638	1.96	6.18E‐04	ATP‐citrate synthase	✓	✓	✗	✗	✗	✓
AL1A1_RAT	P51647	1.86	1.16E‐03	Retinal dehydrogenase 1	✓	✓	✗	✗	✗	✗
6PGD_RAT	P85968	1.80	3.56E‐07	6‐phosphogluconate dehydrogenase, decarboxylating	✓	✗	✗	✗	✓	✗
AL1A7_RAT	P13601	1.76	1.89E‐02	Aldehyde dehydrogenase, cytosolic 1	✓	✗	✗	✗	✗	✗
MARE2_RAT	Q3B8Q0	1.61	1.88E‐02	Microtubule‐associated protein RP/EB family member 2	✗	✗	✗	✗	✗	✗
GSTK1_RAT	P24473	1.60	1.28E‐02	Glutathione S‐transferase kappa 1	✓	✗	✗	✗	✗	✗
GSTT1_RAT	Q01579	1.47	3.89E‐02	Glutathione S‐transferase theta‐1	✓	✗	✗	✗	✗	✗
FAS_RAT	P12785	1.34	2.20E‐03	Fatty acid synthase	✓	✓	✗	✗	✗	✗
ACSL5_RAT	O88813	1.32	8.96E‐03	Long‐chain‐fatty‐acid–CoA ligase 5	✓	✓	✗	✗	✗	✗
CP2A1_RAT	P11711	1.31	1.36E‐02	Cytochrome P450 2A1	✓	✓	✗	✗	✗	✗
CMBL_RAT	Q7TP52	1.29	1.13E‐02	Carboxymethylenebutenolidase homolog	✓	✗	✗	✗	✗	✗
DEST_RAT	Q7M0E3	1.24	1.28E‐02	Destrin	✗	✗	✗	✗	✗	✗
TBA1C_RAT	Q6AYZ1	1.23	9.45E‐03	Tubulin alpha‐1C chain	✓	✗	✗	✗	✗	✗
VAT1_RAT	Q3MIE4	1.18	8.20E‐03	Synaptic vesicle membrane protein VAT‐1 homolog	✓	✗	✗	✗	✗	✗
RL9_RAT	P17077	1.04	3.72E‐02	60S ribosomal protein L9	✗	✗	✗	✗	✗	✗
PUR9_RAT	O35567	0.97	1.14E‐02	Bifunctional purine biosynthesis protein PURH	✓	✗	✗	✗	✗	✗
MIF_RAT	P30904	0.94	1.23E‐04	Macrophage migration inhibitory factor	✓	✓	✗	✗	✗	✗
DHPR_RAT	P11348	0.92	4.00E‐02	Dihydropteridine reductase	✓	✗	✗	✗	✗	✗
PABP1_RAT	Q9EPH8	0.92	1.86E‐04	Polyadenylate‐binding protein 1	✗	✗	✗	✗	✗	✗
TERA_RAT	P46462	0.89	1.93E‐02	Transitional endoplasmic reticulum ATPase	✓	✗	✗	✗	✗	✗
SYDC_RAT	P15178	0.85	1.31E‐02	Aspartate–tRNA ligase, cytoplasmic	✓	✗	✗	✗	✗	✗
EF1A1_RAT	P62630	0.80	6.06E‐03	Elongation factor 1‐alpha 1	✓	✗	✗	✗	✗	✗
SAR1B_RAT	Q5HZY2	0.79	1.20E‐02	GTP‐binding protein SAR1b	✗	✗	✗	✗	✗	✗
PGK1_RAT	P16617	0.77	9.19E‐03	Phosphoglycerate kinase 1	✓	✗	✗	✗	✓	✗
LPP60_RAT	O88202	0.75	4.57E‐02	60 kDa lysophospholipase	✓	✓	✗	✗	✗	✗
EF1D_RAT	Q68FR9	0.72	4.02E‐02	Elongation factor 1‐delta	✗	✗	✗	✗	✗	✗
AL1L1_RAT	P28037	0.69	6.91E‐03	Cytosolic 10‐formyltetrahydrofolate dehydrogenase	✓	✗	✗	✗	✗	✗
GCKR_RAT	Q07071	0.65	1.03E‐02	Glucokinase regulatory protein	✗	✗	✗	✗	✗	✗
ACACA_RAT	P11497	0.63	2.19E‐02	Acetyl‐CoA carboxylase 1	✓	✓	✗	✗	✗	✗
PH4H_RAT	P04176	0.61	4.92E‐02	Phenylalanine‐4‐hydroxylase	✓	✗	✗	✗	✗	✗
RS4X_RAT	P62703	0.59	3.99E‐03	40S ribosomal protein S4, X isoform	✗	✗	✗	✗	✗	✗
DHB4_RAT	P97852	−0.63	2.04E‐02	Peroxisomal multifunctional enzyme type 2	✗	✗	✗	✗	✗	✗
RAB1A_RAT	Q6NYB7	−0.65	1.48E‐02	Ras‐related protein Rab‐1A	✗	✗	✗	✗	✗	✗
HS71A_RAT	P0DMW0	−0.66	3.37E‐02	Heat shock 70 kDa protein 1A	✗	✗	✓	✗	✗	✗
MET7B_RAT	Q562C4	−0.68	1.04E‐02	Methyltransferase‐like protein 7B	✗	✗	✗	✗	✗	✗
DYHC1_RAT	P38650	−0.70	4.05E‐02	Cytoplasmic dynein 1 heavy chain 1	✗	✗	✗	✗	✗	✗
UGDH_RAT	O70199	−0.74	2.43E‐02	UDP‐glucose 6‐dehydrogenase	✗	✗	✗	✗	✗	✗
ENPL_RAT	Q66HD0	−0.75	2.77E‐02	Endoplasmin	✗	✗	✗	✗	✗	✗
XPP1_RAT	O54975	−0.75	2.38E‐02	Xaa‐Pro aminopeptidase 1	✗	✗	✗	✗	✗	✗
NAKD2_RAT	Q1HCL7	−0.76	4.06E‐02	NAD kinase 2, mitochondrial	✗	✗	✓	✗	✗	✗
CAP1_RAT	Q08163	−0.80	2.42E‐03	Adenylyl cyclase‐associated protein 1	✗	✗	✗	✗	✗	✗
PTTG_RAT	Q6P767	−0.82	1.29E‐02	Pituitary tumor‐transforming gene 1 protein‐interacting protein	✗	✗	✗	✗	✗	✗
ANXA6_RAT	P48037	−0.82	3.72E‐02	Annexin A6	✗	✗	✓	✗	✗	✗
RAP1B_RAT	Q62636	−0.83	5.27E‐03	Ras‐related protein Rap‐1b	✗	✗	✗	✗	✗	✗
RL18_RAT	P12001	−0.89	1.86E‐02	60S ribosomal protein L18	✗	✗	✗	✗	✗	✗
ACOX3_RAT	Q63448	−0.90	2.16E‐03	Peroxisomal acyl‐coenzyme A oxidase 3	✗	✗	✓	✗	✗	✗
CH60_RAT	P63039	−0.91	5.12E‐03	60 kDa heat shock protein, mitochondrial	✗	✗	✓	✗	✗	✗
RL32_RAT	P62912	−0.91	3.49E‐02	60S ribosomal protein L32	✗	✗	✗	✗	✗	✗
CALR_RAT	P18418	−0.93	1.95E‐02	Calreticulin	✗	✗	✗	✗	✗	✗
AL3A2_RAT	P30839	−0.93	1.80E‐02	Fatty aldehyde dehydrogenase	✗	✗	✓	✗	✗	✗
GNAI2_RAT	P04897	−0.96	1.36E‐02	Guanine nucleotide‐binding protein G(i) subunit alpha‐2	✗	✗	✗	✗	✗	✗
HACL1_RAT	Q8CHM7	−0.99	6.17E‐03	2‐hydroxyacyl‐CoA lyase 1	✗	✗	✗	✗	✗	✗
GRP78_RAT	P06761	−1.02	7.38E‐03	78 kDa glucose‐regulated protein	✗	✗	✓	✗	✗	✗
GBLP_RAT	P63245	−1.02	2.00E‐02	Guanine nucleotide‐binding protein subunit beta‐2‐like 1	✗	✗	✗	✗	✗	✗
TRFE_RAT	P12346	−1.02	7.94E‐03	Serotransferrin	✗	✗	✗	✗	✗	✗
PP1A_RAT	P62138	−1.02	3.58E‐03	Serine/threonine‐protein phosphatase PP1‐alpha catalytic subunit	✗	✗	✗	✗	✗	✗
UD2B2_RAT	P08541	−1.03	1.65E‐02	UDP‐glucuronosyltransferase 2B2	✗	✗	✗	✗	✗	✗
PRS7_RAT	Q63347	−1.04	1.83E‐02	26S protease regulatory subunit 7	✗	✗	✗	✗	✗	✗
RS8_RAT	P62243	−1.04	6.54E‐03	40S ribosomal protein S8	✗	✗	✗	✗	✗	✗
RHOA_RAT	P61589	−1.04	6.21E‐04	Transforming protein RhoA	✗	✗	✓	✗	✗	✗
RL15_RAT	P61314	−1.05	4.06E‐03	60S ribosomal protein L15	✗	✗	✗	✗	✗	✗
TPP2_RAT	Q64560	−1.08	6.76E‐04	Tripeptidyl‐peptidase 2	✗	✗	✗	✗	✗	✗
MMSA_RAT	Q02253	−1.08	2.18E‐02	Methylmalonate‐semialdehyde dehydrogenase [acylating], mitochondrial	✗	✗	✓	✗	✗	✗
PECR_RAT	Q9WVK3	−1.09	1.14E‐02	Peroxisomal trans‐2‐enoyl‐CoA reductase	✗	✗	✓	✗	✗	✗
ST1E1_RAT	P52844	−1.13	7.95E‐03	Estrogen sulfotransferase, isoform 1	✗	✗	✗	✗	✗	✗
MDHM_RAT	P04636	−1.13	1.22E‐02	Malate dehydrogenase, mitochondrial	✗	✗	✓	✓	✗	✗
PON1_RAT	P55159	−1.15	6.49E‐03	Serum paraoxonase/arylesterase 1	✗	✗	✗	✗	✗	✗
NDUAA_RAT	Q561S0	−1.15	4.53E‐02	NADH dehydrogenase [ubiquinone] 1 alpha subcomplex subunit 10, mitochondrial	✗	✗	✗	✓	✗	✗
ETFD_RAT	Q6UPE1	−1.15	2.07E‐02	Electron transfer flavoprotein‐ubiquinone oxidoreductase, mitochondrial	✗	✗	✓	✓	✗	✗
GYS2_RAT	P17625	−1.15	3.23E‐04	Glycogen [starch] synthase, liver	✗	✗	✗	✗	✗	✗
SPP24_RAT	Q62740	−1.16	4.43E‐02	Secreted phosphoprotein 24	✗	✗	✗	✗	✗	✗
PDC6I_RAT	Q9QZA2	−1.18	2.80E‐03	Programmed cell death 6‐interacting protein	✗	✗	✗	✗	✗	✗
CP2C7_RAT	P05179	−1.18	3.45E‐02	Cytochrome P450 2C7	✗	✗	✗	✗	✗	✗
HMCS2_RAT	P22791	−1.18	2.69E‐02	Hydroxymethylglutaryl‐CoA synthase, mitochondrial	✗	✗	✓	✗	✗	✗
SDHA_RAT	Q920L2	−1.22	1.72E‐02	Succinate dehydrogenase [ubiquinone] flavoprotein subunit, mitochondrial	✗	✗	✓	✓	✗	✗
CLUS_RAT	P05371	−1.22	2.37E‐02	Clusterin	✗	✗	✓	✗	✗	✗
FIBG_RAT	P02680	−1.32	4.72E‐02	Fibrinogen gamma chain	✗	✗	✗	✗	✗	✗
MGST1_RAT	P08011	−1.33	3.29E‐03	Microsomal glutathione S‐transferase 1	✗	✗	✓	✗	✗	✗
PDIA4_RAT	P38659	−1.34	1.49E‐03	Protein disulfide‐isomerase A4	✗	✗	✗	✗	✗	✗
BDH_RAT	P29147	−1.34	2.93E‐02	D‐beta‐hydroxybutyrate dehydrogenase, mitochondrial	✗	✗	✓	✗	✗	✗
CHDH_RAT	Q6UPE0	−1.35	3.82E‐02	Choline dehydrogenase, mitochondrial	✗	✗	✓	✗	✗	✗
S22A7_RAT	Q5RLM2	−1.35	1.24E‐02	Solute carrier family 22 member 7	✗	✗	✓	✗	✗	✗
ST1C1_RAT	P50237	−1.36	1.66E‐02	Sulfotransferase 1C1	✗	✗	✗	✗	✗	✗
RL27A_RAT	P18445	−1.36	1.89E‐03	60S ribosomal protein L27a	✗	✗	✗	✗	✗	✗
RS25_RAT	P62853	−1.38	3.23E‐02	40S ribosomal protein S25	✗	✗	✗	✗	✗	✗
THIM_RAT	P13437	−1.38	2.14E‐02	3‐ketoacyl‐CoA thiolase, mitochondrial	✗	✗	✓	✗	✗	✗
SARDH_RAT	Q64380	−1.41	2.98E‐02	Sarcosine dehydrogenase, mitochondrial	✗	✗	✓	✗	✗	✗
SAC1_RAT	Q9ES21	−1.41	2.13E‐03	Phosphatidylinositide phosphatase SAC1	✗	✗	✗	✗	✗	✗
CPSM_RAT	P07756	−1.41	2.28E‐02	Carbamoyl‐phosphate synthase [ammonia], mitochondrial	✗	✗	✗	✗	✗	✗
CATA_RAT	P04762	−1.43	5.15E‐04	Catalase	✗	✗	✓	✗	✗	✗
AL4A1_RAT	P0C2 × 9	−1.49	3.05E‐02	Delta‐1‐pyrroline‐5‐carboxylate dehydrogenase, mitochondrial	✗	✗	✓	✗	✗	✗
ACON_RAT	Q9ER34	−1.49	3.67E‐02	Aconitate hydratase, mitochondrial	✗	✗	✓	✓	✗	✗
GTR2_RAT	P12336	−1.52	3.16E‐04	Solute carrier family 2, facilitated glucose transporter member 2	✗	✗	✗	✗	✗	✗
IVD_RAT	P12007	−1.53	2.32E‐03	Isovaleryl‐CoA dehydrogenase, mitochondrial	✗	✗	✓	✗	✗	✗
PLMN_RAT	Q01177	−1.53	1.10E‐02	Plasminogen	✗	✗	✗	✗	✗	✗
AGT2_RAT	Q64565	−1.53	9.53E‐03	Alanine–glyoxylate aminotransferase 2, mitochondrial	✗	✗	✓	✗	✗	✗
MVP_RAT	Q62667	−1.54	2.02E‐03	Major vault protein	✗	✗	✗	✗	✗	✗
PBLD_RAT	Q68G31	−1.55	3.84E‐02	Phenazine biosynthesis‐like domain‐containing protein	✗	✗	✗	✗	✗	✗
ACADM_RAT	P08503	−1.58	1.39E‐02	Medium‐chain specific acyl‐CoA dehydrogenase, mitochondrial	✗	✗	✓	✗	✗	✗
H17B6_RAT	O54753	−1.61	1.53E‐03	17‐beta‐hydroxysteroid dehydrogenase type 6	✗	✗	✓	✗	✗	✗
EIF3E_RAT	Q641 × 8	−1.62	9.03E‐03	Eukaryotic translation initiation factor 3 subunit E	✗	✗	✗	✗	✗	✗
SBP1_RAT	Q8VIF7	−1.64	4.77E‐02	Selenium‐binding protein 1	✗	✗	✗	✗	✗	✗
CP2DA_RAT	P12939	−1.66	1.97E‐03	Cytochrome P450 2D10	✗	✗	✗	✗	✗	✗
COR1A_RAT	Q91ZN1	−1.66	2.44E‐02	Coronin‐1A	✗	✗	✗	✗	✗	✗
LG3BP_RAT	O70513	−1.68	3.17E‐02	Galectin‐3‐binding protein	✗	✗	✗	✗	✗	✗
MCCA_RAT	Q5I0C3	−1.70	1.29E‐02	Methylcrotonoyl‐CoA carboxylase subunit alpha, mitochondrial	✗	✗	✓	✗	✗	✗
CP2D1_RAT	P10633	−1.71	9.34E‐04	Cytochrome P450 2D1	✗	✗	✓	✗	✗	✗
AT1A1_RAT	P06685	−1.75	2.41E‐04	Sodium/potassium‐transporting ATPase subunit alpha‐1	✗	✗	✗	✗	✗	✗
CP2CN_RAT	P24470	−1.75	8.02E‐03	Cytochrome P450 2C23	✗	✗	✗	✗	✗	✗
PDIA3_RAT	P11598	−1.75	5.38E‐05	Protein disulfide‐isomerase A3	✗	✗	✓	✗	✗	✗
PCCA_RAT	P14882	−1.77	1.95E‐03	Propionyl‐CoA carboxylase alpha chain, mitochondrial	✗	✗	✓	✗	✗	✗
ANGL4_RAT	Q6TMA8	−1.77	3.93E‐03	Angiopoietin‐related protein 4	✗	✗	✗	✗	✗	✗
HMGCL_RAT	P97519	−1.78	1.01E‐03	Hydroxymethylglutaryl‐CoA lyase, mitochondrial	✗	✗	✓	✗	✗	✗
SDC4_RAT	P34901	−1.78	1.17E‐04	Syndecan‐4	✗	✗	✗	✗	✗	✗
CP2DQ_RAT	P10634	−1.79	1.03E‐03	Cytochrome P450 2D26	✗	✗	✗	✗	✗	✗
PYC_RAT	P52873	−1.82	1.83E‐03	Pyruvate carboxylase, mitochondrial	✗	✗	✓	✗	✗	✗
RS27A_RAT	P62982	−1.84	8.94E‐04	Ubiquitin‐40S ribosomal protein S27a	✗	✗	✗	✗	✗	✗
RDH2_RAT	P50170	−1.86	4.16E‐04	Retinol dehydrogenase 2	✗	✗	✗	✗	✗	✗
ACOX2_RAT	P97562	−1.87	3.22E‐02	Peroxisomal acyl‐coenzyme A oxidase 2	✗	✗	✓	✗	✗	✗
HYEP_RAT	P07687	−1.88	5.86E‐03	Epoxide hydrolase 1	✗	✗	✗	✗	✗	✗
IDHP_RAT	P56574	−1.88	9.46E‐03	Isocitrate dehydrogenase [NADP], mitochondrial	✗	✗	✓	✓	✗	✗
UDB17_RAT	P08542	−1.88	3.09E‐04	UDP‐glucuronosyltransferase 2B17	✗	✗	✗	✗	✗	✗
MOT1_RAT	P53987	−1.95	8.53E‐04	Monocarboxylate transporter 1	✗	✗	✗	✓	✗	✗
CYB5_RAT	P00173	−1.96	3.20E‐03	Cytochrome b5	✗	✗	✓	✗	✗	✗
CP2D4_RAT	Q64680	−2.00	9.96E‐04	Cytochrome P450 2D4	✗	✗	✓	✗	✗	✗
ALDH2_RAT	P11884	−2.03	6.31E‐03	Aldehyde dehydrogenase, mitochondrial	✗	✗	✓	✗	✗	✗
A1M_RAT	Q63041	−2.07	1.11E‐02	Alpha‐1‐macroglobulin	✗	✗	✗	✗	✗	✗
RDH7_RAT	P55006	−2.12	6.66E‐04	Retinol dehydrogenase 7	✗	✗	✗	✗	✗	✗
UDB15_RAT	P36511	−2.16	3.72E‐03	UDP‐glucuronosyltransferase 2B15	✗	✗	✗	✗	✗	✗
PCCB_RAT	P07633	−2.17	1.05E‐02	Propionyl‐CoA carboxylase beta chain, mitochondrial	✗	✗	✓	✗	✗	✗
BASI_RAT	P26453	−2.17	3.95E‐04	Basigin	✗	✗	✓	✓	✗	✗
MBL2_RAT	P08661	−2.21	1.40E‐03	Mannose‐binding protein C	✗	✗	✗	✗	✗	✗
CP2CB_RAT	P08683	−2.52	1.35E‐04	Cytochrome P450 2C11	✗	✗	✗	✗	✗	✗
CD81_RAT	Q62745	−2.53	1.05E‐02	CD81 antigen	✗	✗	✗	✗	✗	✗
ITB1_RAT	P49134	−2.54	9.59E‐03	Integrin beta‐1	✗	✗	✗	✗	✗	✗
PGRC1_RAT	P70580	−2.56	2.82E‐04	Membrane‐associated progesterone receptor component 1	✗	✗	✗	✗	✗	✗
DHE3_RAT	P10860	−2.62	4.55E‐04	Glutamate dehydrogenase 1, mitochondrial	✗	✗	✓	✗	✗	✗
ALBU_RAT	P02770	−2.73	4.69E‐03	Serum albumin	✗	✗	✗	✗	✗	✗
SO1A4_RAT	O35913	−2.97	7.51E‐04	Solute carrier organic anion transporter family member 1A4	✗	✗	✗	✗	✗	✗
4F2_RAT	Q794F9	−3.03	3.32E‐04	4F2 cell‐surface antigen heavy chain	✗	✗	✗	✗	✗	✗
LAMP1_RAT	P14562	−4.40	2.16E‐03	Lysosome‐associated membrane glycoprotein 1	✗	✗	✗	✗	✗	✗

Together, these results suggest a functional role of hepatocyte‐derived EVs in glucose and lipid metabolisms.

### 3.4 Metabolic effects of hepatocyte‐secreted EVs on adipocytes

**FIGURE 6:**
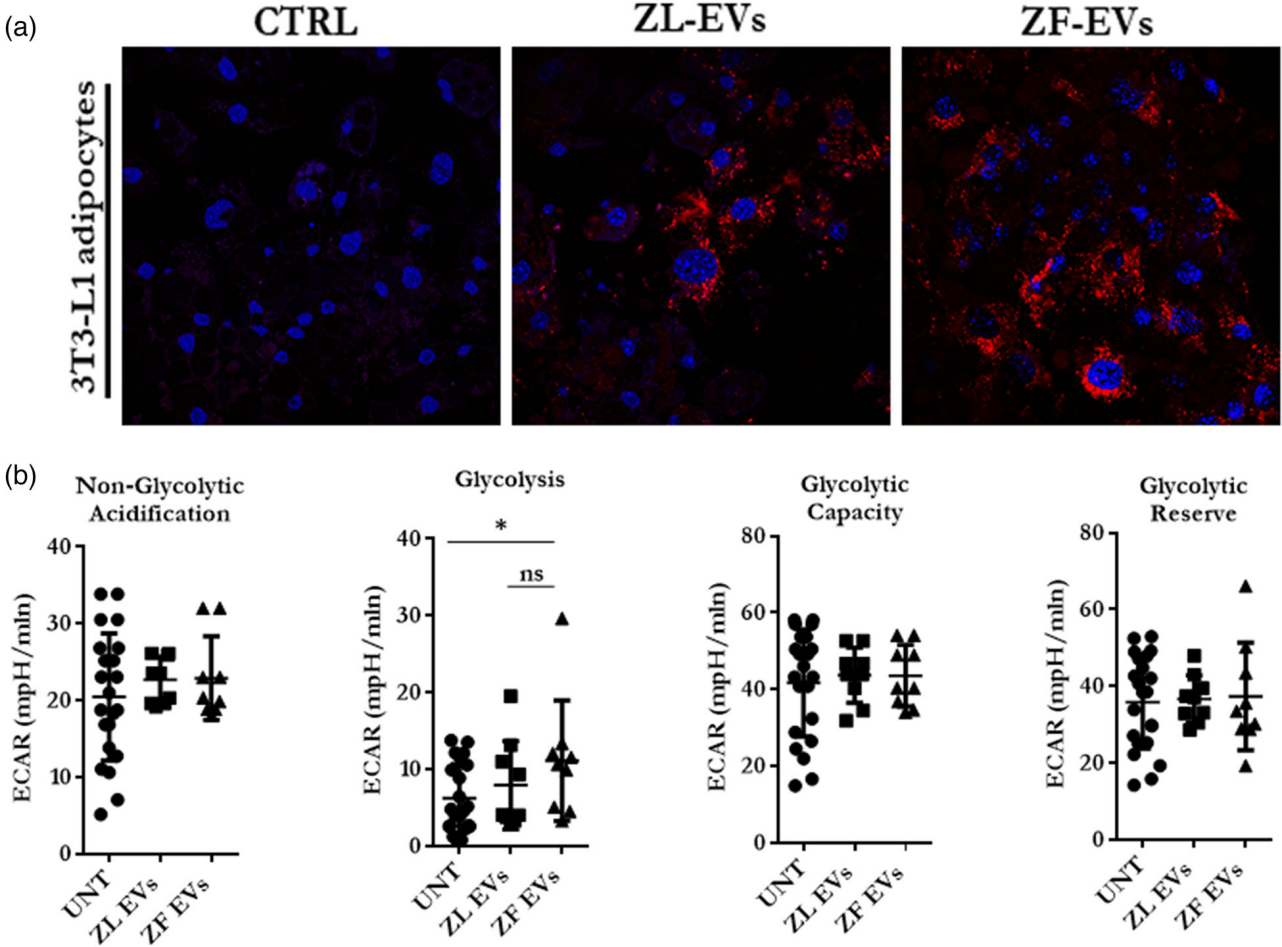
Cellular uptake and energetic effect of hepatic‐EVs on 3T3‐L1 adipocytes. (a) Confocal microscopy uptake analysis of EV‐treated 3T3‐L1 adipocytes. Cells were incubated in the presence of 10 μg of Dil‐labelled ZL‐EVs and ZF‐EVs. CTRL corresponds to 3T3‐L1 adipocytes incubated with DiI containing media after ultracentrifugation. Nucleus are stained with DAPI. (b) Individual parameters of glycolysis stress test on EV‐treated (10 μg) 3T3‐L1 adipocytes, *n* = 3; One‐way ANOVA (*F*(2, 37) = 2.426, *p* = 0.1023)

Given the higher abundance of proteins involved in lipid metabolism in ZF hepatocyte‐ derived EVs, we next investigated the impact of these vesicles on the adipocyte's metabolism using fully differentiated 3T3‐L1 cells. First, to confirm that the rat hepatocyte‐derived EVs were internalized by the mouse 3T3‐L1 adipocytes, we performed uptake assays in which the adipocytes were incubated during 16 h in the presence of DiI‐labelled EVs secreted by ZL‐ or ZF‐primary hepatocytes, and subsequently cells were analysed by confocal microscopy. As can be observed in representative confocal images (Figure 6a), the adipocytes internalized both EVs.

The proteomic analysis revealed that most differentially expressed proteins in ZF‐EVs, were associated with glycolysis and PPP. Thus, in functional experiments we analysed whether these EVs were able to modulate 3T3‐L1 adipocyte's bioenergetics. Glycolysis stress test revealed that ZF‐EVs significantly increased the glycolytic rate of 3T3‐L1 adipocytes as compared to the control, indicating possible transfer of metabolic phenotype (Figure 6b).

**FIGURE 7:**
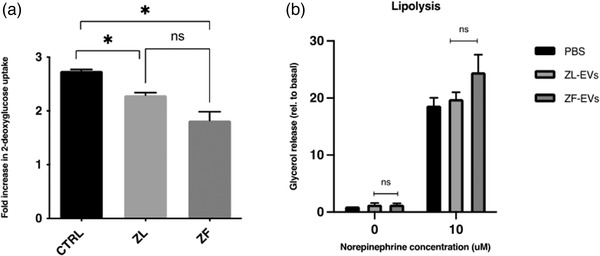
Effect of hepatic‐EVs on 3T3‐L1 adipocytes glucose uptake and lipolysis. (a) Quantification of the fold increase in 2‐deoxy‐glucose uptake in 3T3‐L1 adipocytes that were incubated in absence or presence 15 μg of EVs from ZL‐ and ZF‐EVs during 24 h. (b) Glycerol release following 2 h stimulation with 10 μM norepinephrine of 3T3‐L1 adipocytes untreated (PBS) or treated for 16 h with 5 μg EVs from ZL or ZF hepatocytes. Data is shown relative to untreated, unstimulated cells. *n* = 2; error bars = SD calculated as *s* = |x2−x1|/√2. The *p* values were denoted as follows: ns *p* > 0.05, * *p* ≤ 0.05, ** *p* ≤ 0.01, *** *p* ≤ 0.001, **** *p* ≤ 0.0001

We next looked at insulin sensitivity in 3T3‐L1 adipocytes following treatment with hepatic‐EVs. Insulin‐stimulated glucose uptake was measured following treatment with hepatic‐EVs (Figure 7a). In untreated control adipocytes insulin increased 2‐deoxyglucose uptake by nearly threefold over basal unstimulated cells. Cells treated with EVs from the ZL‐group exhibited a similar response to insulin to that observed in Control cells not treated with EVs. On the contrary, adipocytes treated with ZF‐EVs displayed a significant decrease in insulin‐stimulated glucose uptake (Figure 7a), suggesting that EVs from ZF hepatocytes promote insulin resistance in adipocytes.

We next studied the possible effect of hepatic‐EVs on lipid metabolism in recipient adipocytes. Lipolysis assay was performed in basal conditions or in response to β‐adrenergic stimulation. For this experiment, adipocytes were pretreated with 5 μg of ZL‐ or ZF‐EVs for 16 h, cells were then left untreated or treated with norepinephrine and lipolysis was determined by quantifying free glycerol released to the conditioned medium (Figure 7b). As observed, there was an increase in lipolytic response in the adipocytes treated with ZF‐EVs as compared to the control group.

**FIGURE 8:**
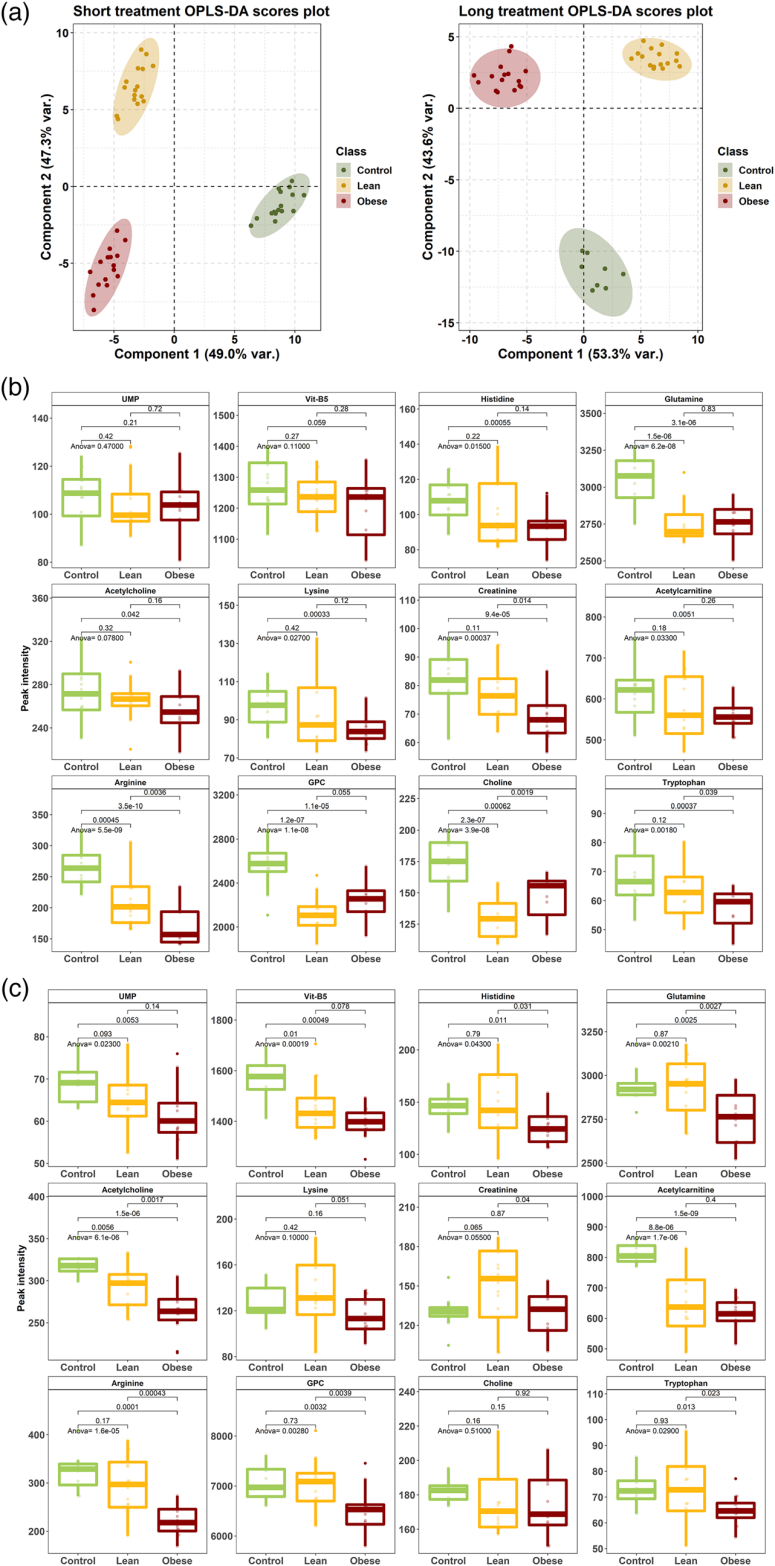
Metabolomics analysis of 3T3‐L1 adipocytes treated with ZL‐ and ZF‐EVs. (a) Scores OPLS‐DA plots for short (1 × 10 μg for 24 h) and long (3 × 10 μg every 24 h) treatments. Samples have been coloured depending on their class (CTRL, ZL, and ZF) in green, orange, and red, respectively. (b) Boxplots summarizing the differences per sample groups and treatment for the 12 identified metabolites in short treatment. (c) Boxplots summarizing the differences per sample groups and treatment for the 12 identified metabolites in long treatment. Adjusted *p*‐values for both ANOVA and pairwise *t*‐tests are indicated in the corresponding group comparisons

Finally, to further explore the metabolic changes elicited by ZL‐ and ZF‐EVs in adipocytes, we performed an untargeted metabolomics analysis of 3T3‐L1 adipocytes following an acute exposure to ZL‐ or ZF‐EVs (short treatment) or three consecutive 24 h‐treatments with EVs (long treatment). We were able to detect 1029 metabolic peaks and supervised multivariate analysis showed a clear cluster discriminating all three sample groups (CTRL, ZL‐ or ZF‐EVs) in both the short and long treatment conditions (Figure 8). The univariate analysis detected in the short treatment 91 and 127 metabolic peaks that were significantly affected by the incubation with ZL‐ and ZF‐EVs, respectively, while in the long treatment 89 metabolic peaks were altered by ZL‐ and ZF‐EVs. From these metabolic peaks, we were able to identify 12 metabolites belonging to different metabolic pathways including vitamin‐B5, UMP, several amino acids, creatinine (urea cycle), choline, acetylcholine and lipid‐related metabolites (GPC, acetylcarnitine). There was a different response in the adipocytes depending on the origin and the concentration of the EVs used (Figure 8b and Table 2). EVs from obese animals had more pronounced effects; the short treatment significantly affected the metabolism of histidine, glutamine, lysine, arginine, tryptophan, creatinine, choline, acetylcholine, GPC and acetylcarnitine. The longer treatment affected UMP and Vitamin‐B5 metabolism. The EVs derived from lean hepatocytes significantly affected glutamine, arginine, GPC and Choline in the single‐dose treatment, whereas the long‐term treatment also altered Vitamin‐B5, acetylcholine, and acetylcarnitine metabolism. Remarkably, histidine and tryptophan levels were specifically altered by EVs derived from obese hepatocytes in both short and long treatments (Table 2).

**TABLE 2:** Analysis of adipocytes metabolites differentially altered by hepatocytes‐secreted EVs obtained from the Zucker model

	Short treatment				Long treatment			
	Non‐obese model (ZL)		Obese model (ZF)		Non‐obese model (ZL)		Obese model (ZF)	
	Log (FC)	*p*‐value	Log (FC)	*p*‐value	Log (FC)	*p*‐value	Log (FC)	*p*‐value
Histidine	−0.10	0.2166	−0.22	0.0005	0.03	0.7933	−0.21	0.0110
Tryptophan	−0.11	0.1247	−0.25	0.0004	0.01	0.9320	−0.17	0.0126
Lysine	−0.07	0.4154	−0.20	0.0003	0.09	0.4157	−0.12	0.1613
Glutamine	−0.15	0.0000	−0.15	0.0000	−0.01	0.8651	−0.10	0.0025
Arginine	−0.32	0.0004	−0.64	0.0000	−0.15	0.1683	−0.56	0.0001
Creatinine	−0.10	0.1069	−0.26	0.0001	0.19	0.0647	−0.01	0.8675
Choline	−0.42	0.0000	−0.23	0.0006	−0.06	0.1648	−0.06	0.1495
Acetyl‐Choline	−0.04	0.3199	−0.10	0.0416	−0.13	0.0056	−0.28	0.0000
Glycerophosphocholine (GPC)	−0.27	0.0000	−0.20	0.0000	−0.01	0.7345	−0.12	0.0032
Acetylcarnitine	−0.09	0.1790	−0.15	0.0051	−0.35	0.0000	−0.40	0.0000
Vit‐B5	−0.04	0.2693	−0.08	0.0588	−0.11	0.0104	−0.17	0.0005
UMP	−0.04	0.4199	−0.06	0.2138	−0.09	0.0934	−0.17	0.0053

Taken together these findings show that hepatic‐EVs significantly affect adipocyte´s metabolism and elicit differential effects based upon the metabolic status of the hepatocytes that produce the EVs.

## 4 DISCUSSION

In obesity, increased caloric intake results in ectopic lipid accumulation in many tissues, including the liver. The so‐called nonalcoholic fatty liver disease (NAFLD) is a crucial feature of obesity and the metabolic syndrome. Hepatic steatosis induces metabolic alterations in hepatocytes, lipotoxicity, cell death, fibrosis and inflammation within the liver, which in turn has systemic effects in other essential metabolic organs. Studies have shown that circulating EVs in humans and in animal models with NAFLD contain biomarkers that serve to evaluate the severity and progression of the disease (Eguchi & Feldstein, 2018).

In this work, we characterized in depth the EVs secreted by cultured primary hepatocytes obtained from Zucker rat, a rodent obesity model that recapitulates many features of the metabolic syndrome, including liver steatosis and insulin‐resistance.

We demonstrate that hepatocytes from obese animals do secrete more EVs and these vesicles exhibit differential physical properties and protein composition from those released by nonobese animals. We also examined the metabolic effects of these EVs in recipient adipose cells. We showed that EVs produced by steatotic hepatocytes alter adipose cell metabolism, causing alterations in cell signalling, glucose lipid and amino acid metabolism, thus effectively contributing to adipocyte cell dysfunction and deranged inter tissue communication seen in obesity and metabolic syndrome.

Our findings show that hepatocytes obtained from obese Zucker animals released more EVs than their lean counterparts. This increase in EV agrees with previous reports showing obesity has been associated with an increase in circulating EVs (Eguchi et al., 2016; Khalyfa et al., 2016; Kranendonk et al., 2014; Lee et al., 2016). Hepatocyte EV production has been shown to change quantitatively and qualitatively in response to cellular stimulation, cellular stress, pharmacological treatments and disease conditions (Conde‐Vancells et al., 2008, 2010; Momen‐Heravi et al., 2015; Palomo et al., 2018; Royo & Falcon‐Perez, 2012) including high fat diet (HFD) (Zhao et al., 2020). In this latter study, Zhao et al., explored the topic of hepatic‐EVs in adipose tissue remodelling in response to HFD. Although there are similarities in the focus of both studies, however, methods employed, such as culture of primary cells and isolation of EVs, and more importantly the experimental models used, are fundamentally different. These crucial factors play a role in the type of EVs that are being studied and characterized and might explain the discrepancy in WB results between both studies. Results published by Zhao et al., show that EVs secreted by the liver following a change in metabolic environment, specifically of lipid overload, caused adipose tissue remodelling. They used a HFD model to induce a rapid lipid overload in mice, which caused observable changes in lipid liver content within hours (acute model). In our study, as far as we are aware for the first time, we used the culture of primary hepatocytes from Zucker rat for the isolation of hepatic‐EVs. Zucker rat model is a very well‐known and well‐characterized genetic model of obesity. Mutation in the leptin receptor gene causes hyperphagia in this rat model and drives the metabolic, prediabetic, consequences, thus being similar to a chronic model of obesity. Moreover, the culture of primary hepatocytes and subsequent isolation of EVs are different to the method used by Zhao et al., which might also contribute to explain the contradiction in biochemistry of EVs between both studies. Our WB results indicate the decrease in expression of exosomal markers in the population of steatotic EVs, as well as decrease in the population of EVs as seen by Nanoparticle Tracking Analysis. On the contrary, Zhao et al., found an increase in the EV‐marker expression HFD group as compared to the control. Thus, these discrepancies could reflect that we are studying different populations of EVs due to differences in culture and isolation times as well as a study of a different model overall. Remarkably, some similarities remain in both studies, notably there is an increase in the EV production by obese primary hepatocytes isolated from mice under HFD or from our prediabetic Zucker rat, which is also a well‐established phenomenon in the field and supports that obesity induced EV secretion in hepatocytes although their composition could be different in acute and chronic stages.

We found EVs obtained from ZF hepatocytes showed a broader range in vesicle size, differential density and proteomic composition. In this regard, we found that EVs from obese animals showed reduced levels of exosomal markers Cd63, Cd81, Flotillin1 and increased levels of Hsp70 and Hsp90. These changes mirrored the pattern observed in the corresponding cellular lysates, corroborating other reports that show EV composition reflects the parental cell (Driedonks et al., 2018; Vagner et al., 2018; Yáñez‐Mó et al., 2015). Some degree of inconsistency between the independent preparations is to be expected due to an individual and animal variability of the primary cultures and subsequently the biochemistry of their secreted EVs. The ratio between the ng of proteins per million of particles is the same between both samples indicative that we might be working with condition‐specific population of EVs. In 2016, Kowal et al. reported subcategories of small‐EVs defined by their degree of enrichment in CD63, CD9, and/or CD81 tetraspanins (Kowal et al., 2016). The increase in HSPs in EVs from obese animals may suggest preferential loading and export of these proteins into EVs. Similar findings have been reported in EVs released in other pathological conditions. For example, Hsp90 has also been exported in EVs produced by cancer cells and recently has been reported to enhance exosome formation (Lauwers et al., 2018; Ono et al., 2018) which would be consistent with our finding of higher abundance of EVs released by the obese hepatocyte group. Interestingly, we detected adiponectin in EVs produced by hepatocytes, exhibiting decreased expression in ZF hepatocytes, consistent with lower adiponectin signalling reported in fatty livers (Combs & Marliss, 2014). The presence of this adipokine is consistent with previous findings of hepatic phenotypic alterations observed during NAFLD progression in humans. These have reported alterations in expression of adiponectin, and PPARγ, a transcription factor involved in the regulation of adiponectin gene, but also of other proteins associated with lipid metabolism and inflammation, including adipocyte protein (aP2), the fatty acid transporter CD36, and proinflammatory cytokines: IL‐6 and IL‐18 (Lakhani et al., 2018).

Our previous proteome studies in EVs obtained from isolated primary hepatocytes had found many enzymes involved in endogenous and xenobiotic metabolism (Palomo et al., 2018; Yáñez‐Mó et al., 2015). In this study we extended these findings and detected an increase in the presence of glycolytic enzymes: G6PDH and 6PGD, predominantly present in the EVs obtained from hepatocytes harvested from obese animals. Glycolytic flux in recipient adipocyte cells showed a slight increase in cells treated with ZF‐treated group with no changes in glycolytic capacity nor the glycolytic reserve, which might indicate more active degradation of glucose through a nonoxidative pathway. This change could be related with consumption of oxygen associated to EVs capture and processing but does not affect the capability of the cell to switch to glycolytic status when OXPHOS is inhibited.

We also detected an enrichment of enzymes involved in de nov*o* lipogenesis^1^ such as acetyl‐CoA carboxylase, G6PD and Fas in ZF derived EVs. Consistent with our findings, another study found these proteins in exosomes from the obesity mouse model ob/ob (Sano et al., 2014). In addition, our data agrees with that of a recent study showing EVs obtained from hepatocytes from high fat fed mice increased triglyceride accumulation in preadipocyte cells and stimulated adipogenesis of undifferentiated cells (Zhao et al., 2020). The authors also detected an increased abundance of Fas in the EVs released by hepatocytes, which we replicated here in our experimental animal model.

Taken together these data suggest that EVs derived from steatotic hepatocytes could transfer glycolytic enzymes and enzymes involved in lipogenesis, which could drive glucose uptake and lipid deposition in the recipient adipose cells. Chronic exposure to these EVs however, induced insulin resistance, as we observed adipocytes‐treated with ZF derived ‐EVs displayed reduced insulin‐stimulated 2‐deoxyglucose uptake compared to cells exposed to control EVs. This may be explained from the increased lipid deposition in adipocytes which results in adipocyte dysfunction and generates local insulin resistance. Healthy adipocytes store excess energy and release it according to the nutritional status for the maintenance of whole‐body energy homeostasis. However, when chronic energy surplus overcomes the buffering capacity of adipocytes, such as in obesity, this results in local insulin resistance as well as lipid dysregulation and local inflammation. Lipid hypertrophy *per se* has been shown to cause insulin resistance in vitro, in 3T3L1 adipocytes independent of inflammation (Kim et al., 2015). Kim et al., showed that chronically treated 3T3L1 adipocytes with MUFA displayed increased intracellular lipid deposition and impaired insulin‐dependent glucose uptake, with reduced translocation of the glucose transporter GLUT4 to the plasma membrane in response to insulin. The effects were independent of the induction of inflammatory pathways or any impairment in the proximal insulin receptor signalling cascade. While the precise molecular mechanisms underpinning this finding are presently unknown, these results agree with our findings presented here and are consistent with findings obtained in vivo. For example, patients with Cushing's syndrome, with excess of circulating glucocorticoids develop central obesity and insulin resistance with suppressed immune responses. Second, findings obtained in knock out mice models, including the reduction of early B cell factor 1 (EBF1) and JNK1 null mice fed a high fat diet, increases adipocyte cell size and provokes insulin resistance but does not influence the inflammatory pathways (Gao et al., 2014). Similar results were obtained in the JNK1 null mice fed a high fat diet (Lee et al., 2011). Further, previous human studies have also demonstrated a strong association between circulating Fas levels, body mass index and insulin resistance (Fernandez‐Real et al., 2010).

Adipocytes treated with ZF‐derived EVs also showed a reduction in overall metabolism and mitochondrial function as evidenced by reduced OCR and lower acylcarnitine levels found in the metabolome discovery. Acylcarnitine has been used as a surrogate marker for mitochondrial activity (Bjørndal et al., 2018). It is therefore reasonable to hypothesize that steatotic‐EVs may mediate phenotypic transfer in healthy cells, propagating the phenotypic changes observed in the parental cells. In ZF rats a previous study showed that due to hepatic fat accumulation, there was an impairment in mitochondrial function, reflecting the loss of oxidative phosphorylation capacity (Teodoro et al., 2006). In agreement with this hypothesis, our proteomic analysis of hepatocyte‐secreted EVs has shown that they reflect the metabolic state of the parental cell, with the majority (>75%) of proteins identified as downregulated in ZF‐EVs being associated with mitochondria. Furthermore, the metabolomics approach revealed interesting changes in adipocyte metabolites that provide additional support to our findings. For example, we found vitamin‐B5, the main precursor for the biosynthesis of coenzyme A which plays a central role in the metabolism of lipids and carbohydrates.

However, the composition of EVs is not a mere copy of cytosolic content; instead, specific molecules are selectively sorted into EVs and the amount and content of EVs do change in response to different stimuli (Villarroya‐Beltri et al., 2016). We postulate that in the case of ZF‐EVs, their production and altered composition may be associated with an altered process of biogenesis, as reflected by the differential presence of exosomal markers detected in these vesicles, including: Rab8, Cd81, Cd63, Tsg101, Aip1/Alix, Flotillin1 and LimpII, and the association of heat shock proteins. This hypothesis however deserves further investigation. To sum up, our study demonstrates that hepatic steatosis significantly alters the biology of secreted EVs and that these vesicles, in turn, elicit metabolic effects in recipient adipose cells.

Lastly, it is important to highlight that although primary hepatocytes are a gold‐standard in vitro system to study hepatocyte biology, there are several limitations to consider. It is widely recognized that cultured hepatocytes are a subject to gradual loss of liver‐specific functions (Auth et al., 1998), which can subsequently influence secreted EVs. In addition, the variability generated by primary cultures is also a concern and we have tried to reduce their impact by analysing more than ten independent paired preparations of primary hepatocytes from ZL and ZF rats. Moreover, techniques routinely used for purification of EVs mainly involve differential ultracentrifugation, and hence, are not selective for any specific type of vesicles, and the preparations could contain other extracellular particles. Finally, although our results show differences in the secreted material between lean and obese hepatocytes, since we are employing bulk techniques for the characterization of our EV preparations, we could not conclude that the differences are because they secrete different subpopulations of extracellular particles. Further investigation will be required to clarify this aspect.

## CONFLICT OF INTERESTS

None.

## Supporting information

Figure S1

Figure S2

Figure S3

Figure S4

Figure S5

Figure S6

Table S1

Table S2

Table S3
