# Supplementary figures and images for: Extracellular vesicles released by steatotic hepatocytes alter adipocyte metabolism
Source: J Extracell Biol. 2022 Mar 15;1(2):e32. doi: 10.1002/jex2.32 (PMC11080919; doi:10.1002/jex2.32)

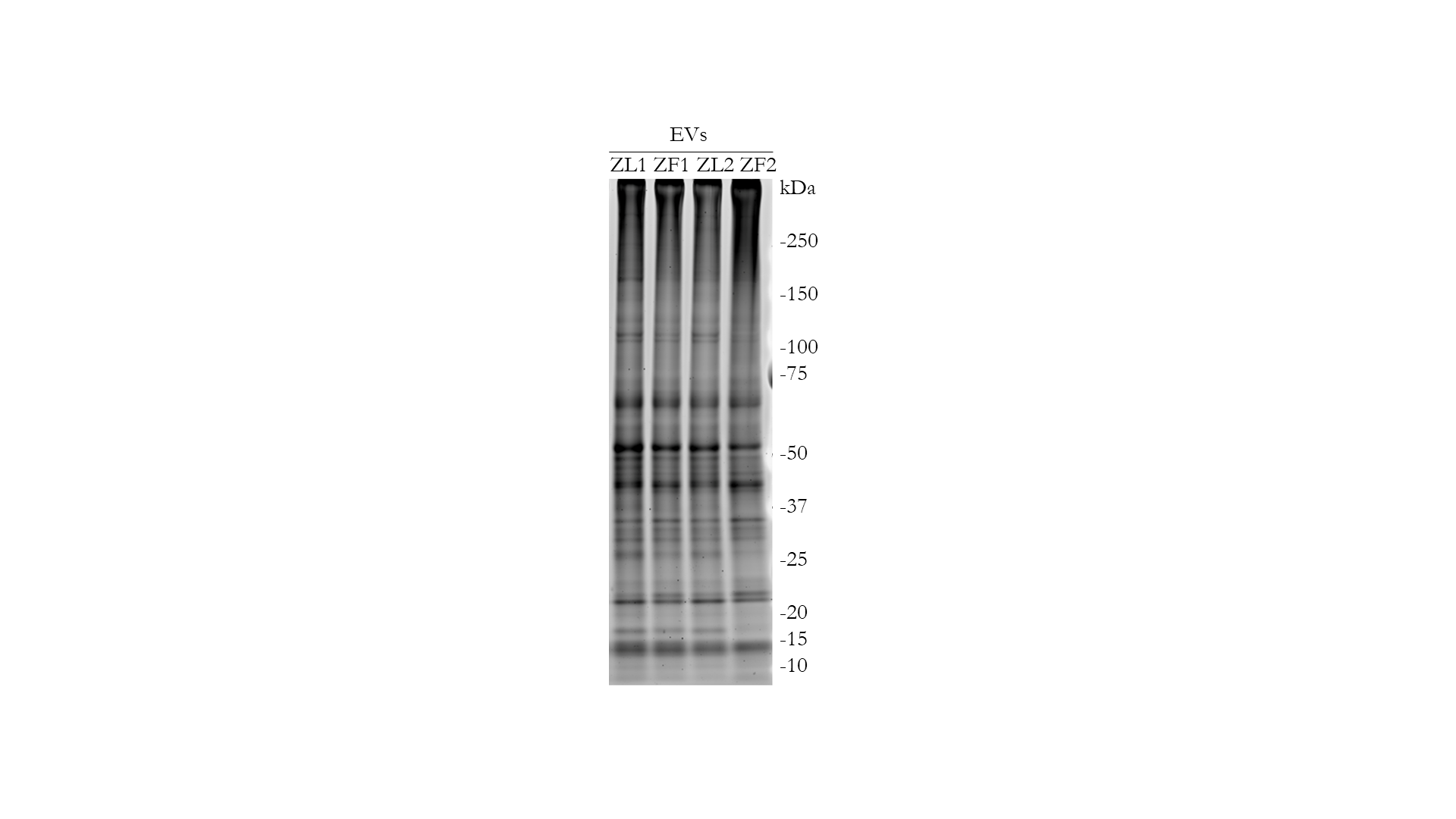

Supplement: Supplementary file 1 — Figure S1 [file JEX2-1-e32-s002.tif]

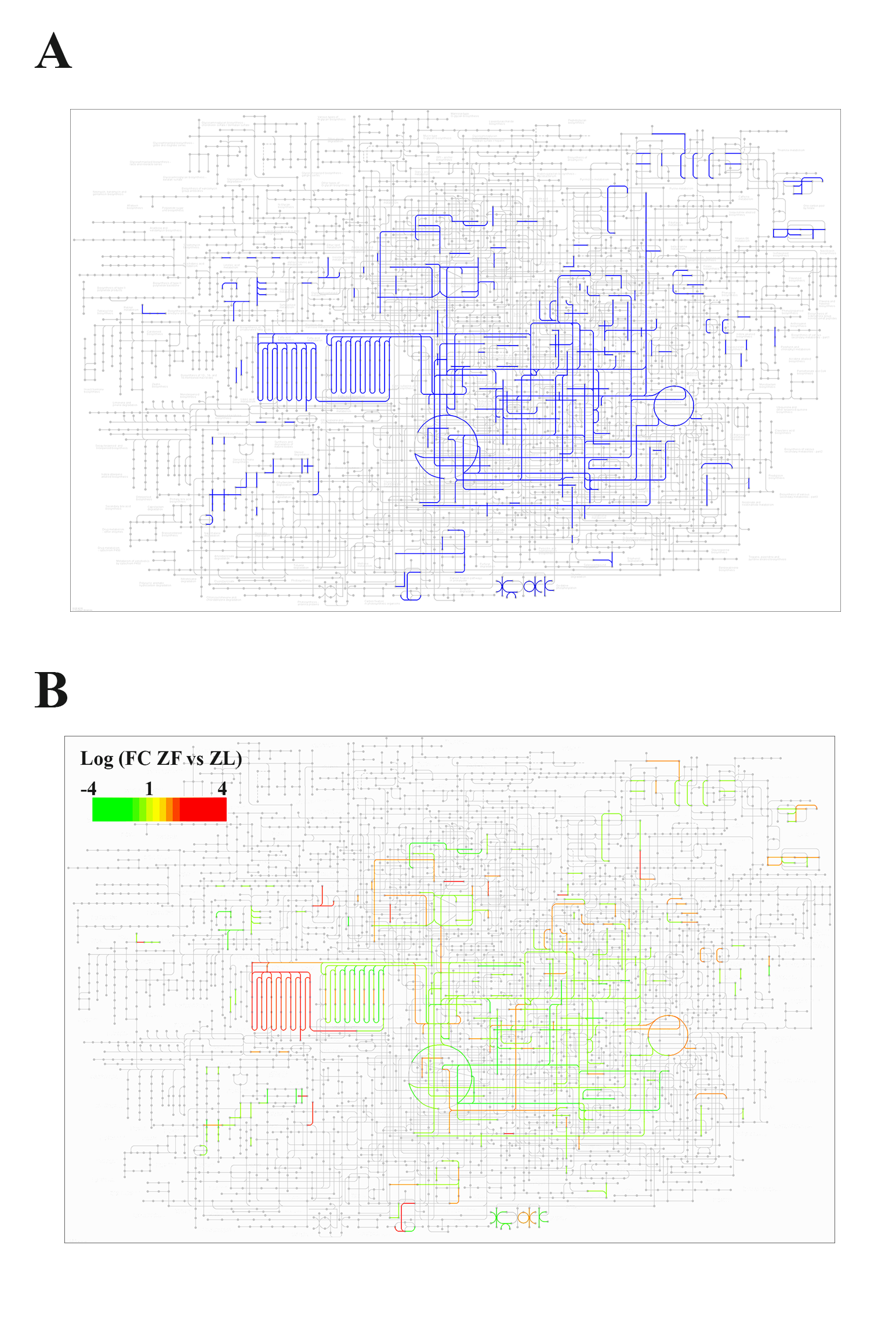

Supplement: Supplementary file 2 — Figure S2 [file JEX2-1-e32-s001.tif]

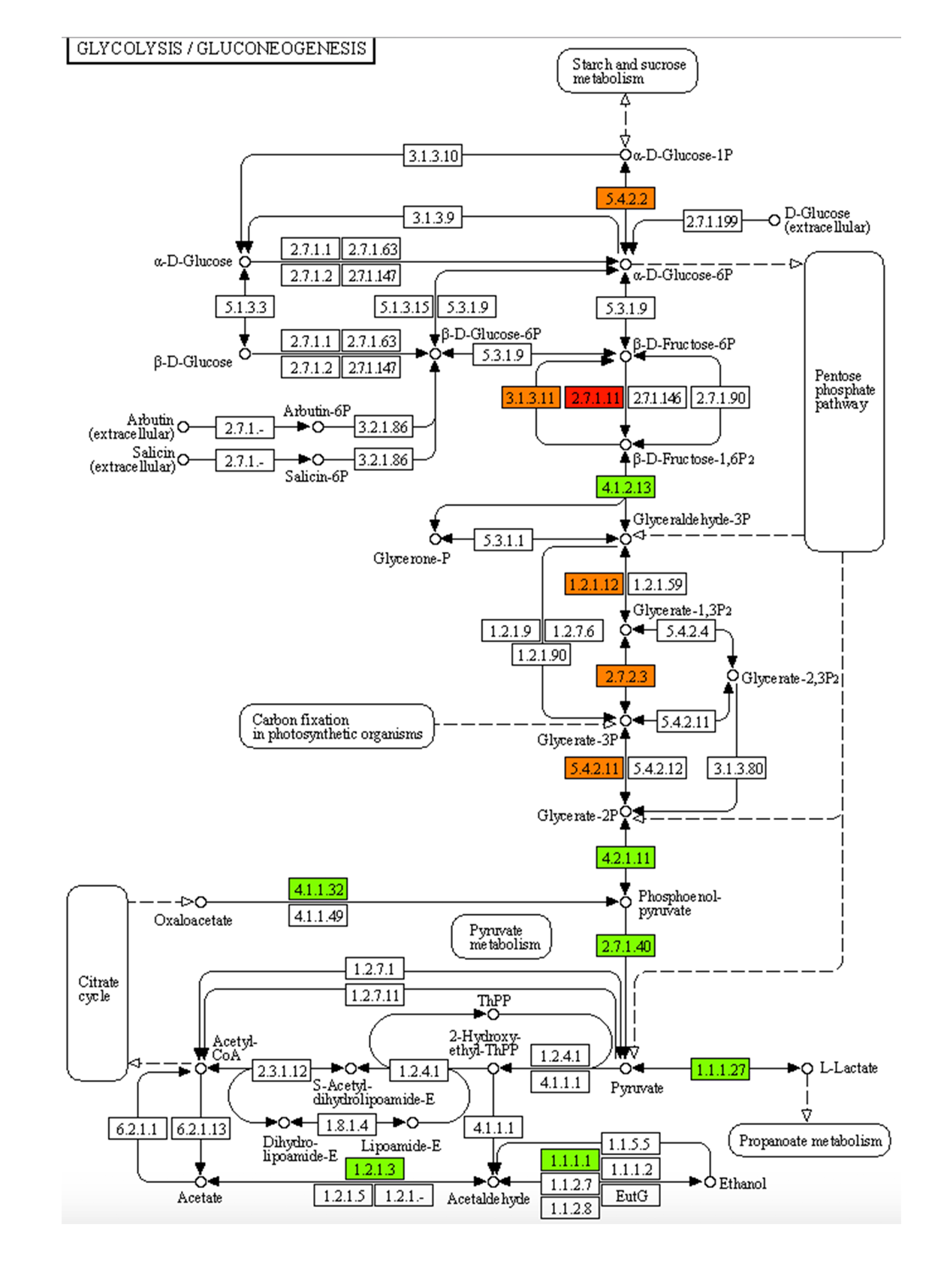

Supplement: Supplementary file 3 — Figure S3 [file JEX2-1-e32-s005.tif]

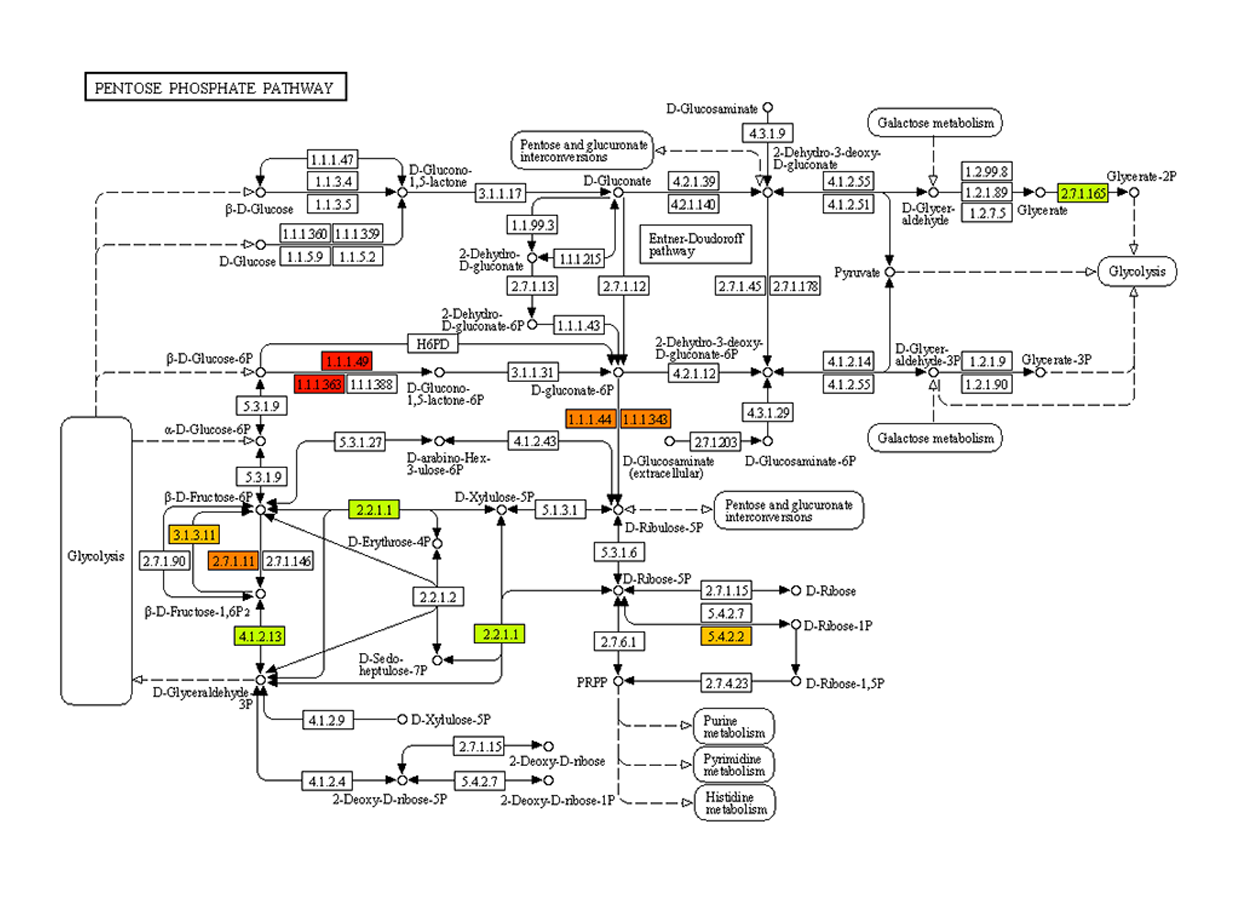

Supplement: Supplementary file 4 — Figure S4 [file JEX2-1-e32-s009.tif]

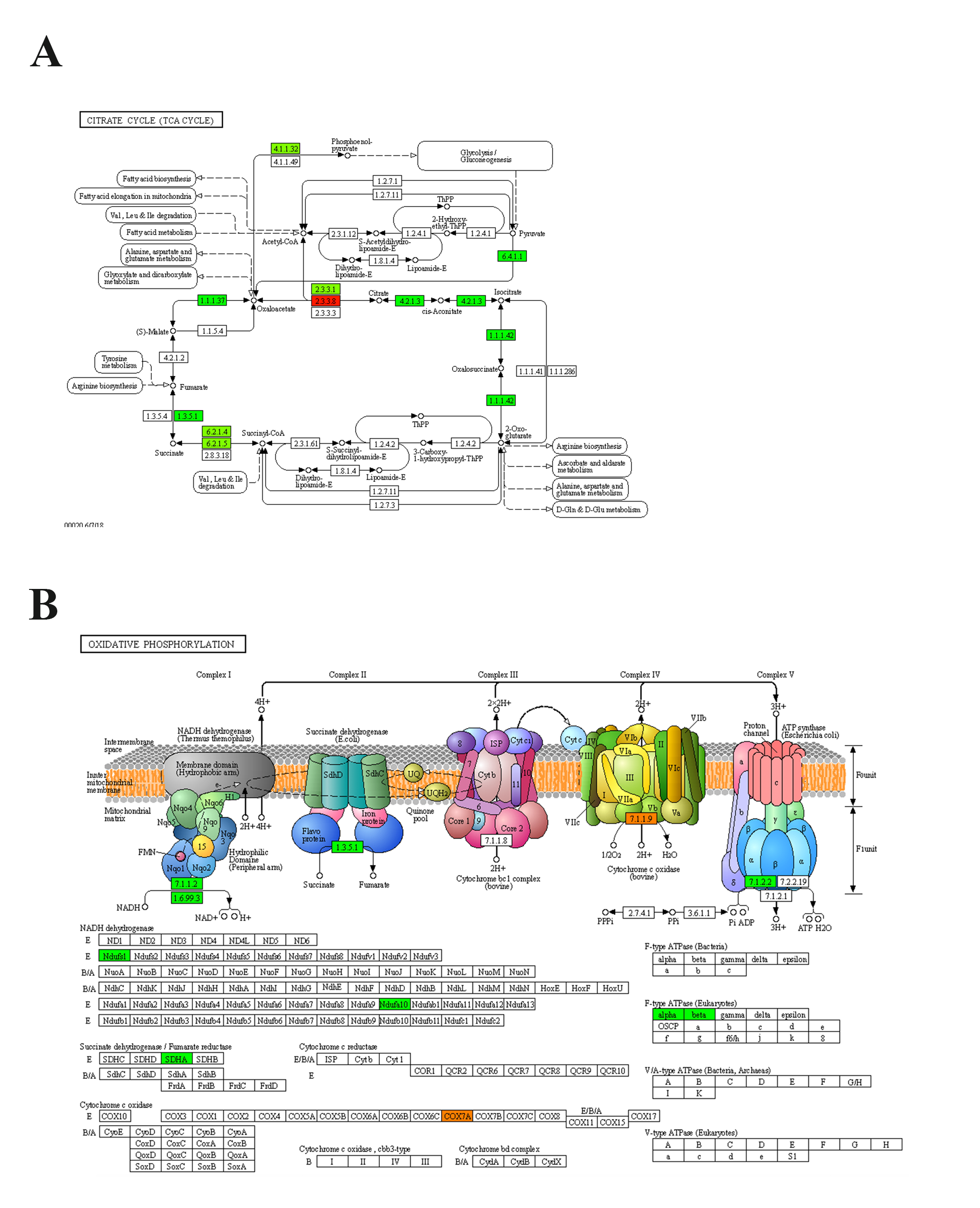

Supplement: Supplementary file 5 — Figure S5 [file JEX2-1-e32-s006.tif]

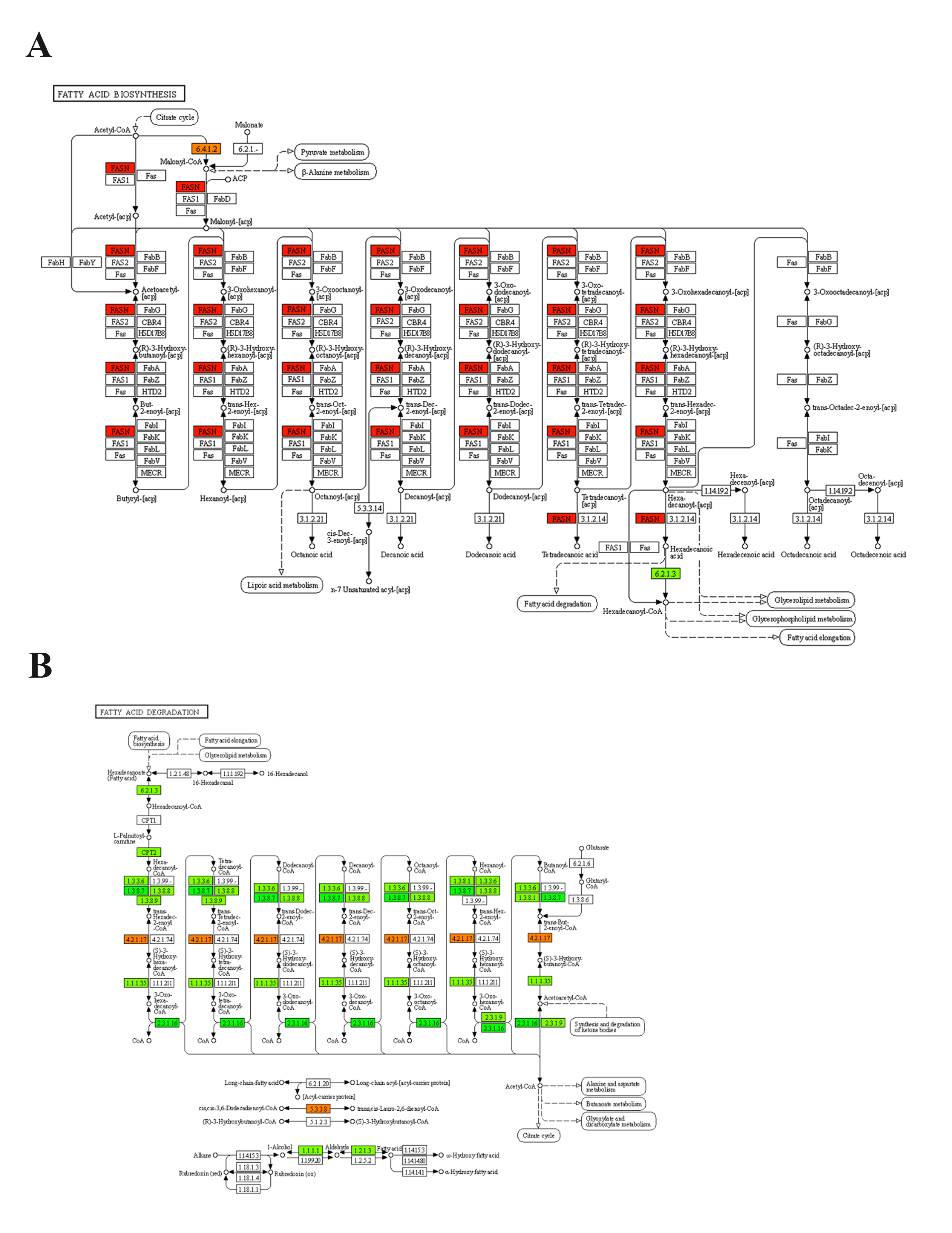

Supplement: Supplementary file 6 — Figure S6 [file JEX2-1-e32-s004.tif]
